# Integrated temporal transcriptional and epigenetic single-cell analysis reveals the intrarenal immune characteristics in an early-stage model of IgA nephropathy during its acute injury

**DOI:** 10.3389/fimmu.2024.1405748

**Published:** 2024-10-18

**Authors:** Chen Xu, Yiwei Zhang, Jian Zhou, Jiangnan Zhang, Hui Dong, Xiangmei Chen, Yi Tian, Yuzhang Wu

**Affiliations:** ^1^ Institute of Immunology, Third Military Medical University (Army Medical University), Chongqing, China; ^2^ The Second Affiliated Hospital, Third Military Medical University (Army Medical University), Chongqing, China; ^3^ Department of Nephrology, Chinese People's Liberation Army (PLA) General Hospital, Chinese People's Liberation Army (PLA) Institute of Nephrology, National Key Laboratory of Kidney Diseases, National Clinical Research Center for Kidney Diseases, Beijing, China; ^4^ Chongqing International Institute for Immunology, Chongqing, China

**Keywords:** IgA nephropathy, immune cells, single-cell RNA-seq, single-cell ATAC-seq, macrophages, CD8^+^ T cells

## Abstract

**Rationale:**

Kidney inflammation plays a crucial role in the pathogenesis of IgA nephropathy (IgAN), yet the specific phenotypes of immune cells involved in disease progression remain incompletely understood. Utilizing joint profiling through longitudinal single-cell RNA-sequencing (scRNAseq) and single-cell assay for transposase-accessible chromatin sequencing (scATACseq) can provide a comprehensive framework for elucidating the development of cell subset diversity and how chromatin accessibility regulates transcription.

**Objective:**

We aimed to characterize the dynamic immune cellular landscape at a high resolution in an early IgAN mouse model with acute kidney injury (AKI).

**Methods and results:**

A murine model was utilized to mimic 3 immunological states –”immune stability (IS), immune activation (IA) and immune remission (IR)” in early human IgAN-associated glomerulopathy during AKI, achieved through lipopolysaccharide (LPS) injection. Urinary albumin to creatinine ratio (UACR) was measured to further validate the exacerbation and resolution of kidney inflammation during this course. Paired scRNAseq and scATACseq analysis was performed on CD45^+^ immune cells isolated from kidney tissues obtained from CTRL (healthy vehicle), IS, IA and IR (4 or 5 mice each). The analyses revealed 7 major cell types and 24 clusters based on 72304 single-cell transcriptomes, allowing for the identification and characterization of various immune cell types within each cluster. Our data offer an impartial depiction of the immunological characteristics, as the proportions of immune cell types fluctuated throughout different stages of the disease. Specifically, these analyses also revealed novel subpopulations, such as a macrophage subset (Nlrp1b Mac) with distinct epigenetic features and a unique transcription factor motif profile, potentially exerting immunoregulatory effects, as well as an early subset of Tex distinguished by their effector and cytolytic potential (CX3CR1^-^transTeff). Furthermore, in order to investigate the potential interaction between immune cells and renal resident cells, we conducted single-cell RNA sequencing on kidney cells obtained from a separate cohort of IS and IA mice without isolating immune cells. These findings underscored the diverse roles played by macrophages and CD8^+^ T cells in maintaining homeostasis of endothelial cells (ECs) under stress.

**Conclusions:**

This study presents a comprehensive analysis of the dynamic changes in immune cell profiles in a model of IgAN, identifying key cell types and their roles and interactions. These findings significantly contribute to the understanding of the pathogenesis of IgAN and may provide potential targets for therapeutic intervention.

## 1 Introduction

IgAN is the most common primary glomerular disease worldwide characterized by the deposition of IgA-containing immune complexes in the mesangium, followed by mesangial hypercellularity and matrix expansion (1–4). Its glomerular histopathology also includes infiltration of inflammatory cells, proliferation of endocapillary, and formation of crescents. IgAN patients show great heterogeneity in their clinical manifestation, ranging from asymptomatic microscopic hematuria to rapidly progressing glomerulonephritis (3, 4). At present, effective targeted therapies are very limited for this tissue-specific autoimmune disease, and 30% to 40% of cases progress to uremia within 20 to 30 years (5).

The infiltration of macrophages and T cells in the glomerulus and/or interstitial compartments has been corroborated to play an initiative role in regulating renal inflammation and contribute to IgAN progression (6, 7). Recently, scRNAseq analyses have unveiled the complexity of cellular phenotypes in both normal and injured human and mouse kidneys, thereby revolutionizing our understanding of kidney disease pathologies from diverse perspectives (8–19). However, the phenotypes of specific immune cell types in the IgAN kidney are not well understood.

Integrated analysis of multimodal single-cell omics data is an emerging tool to describe an unbiased and comprehensive view of cell atlas. For example, Giles et al. demonstrated that scRNAseq data obtained from the LCMV model of CD8^+^ T cell differentiation had less resolution in defining cell identity whereas the paired scATACseq data outperformed in determining cell “fates” (20). Joint profiling by longitudinal scRNAseq and scATACseq can provide a framework for understanding how development of cell subset diversity as well as how chromatin accessibility regulates transcription (16, 21). And immune cells in different pathology contexts may have distinct chromatin accessibility profiles that change as they differentiate.

In the current study we used an early IgAN model with an extra LPS injection to mimic disease exacerbation resulting from mucosal infection and/or a reversible form of AKI, which is frequently observed in clinical practice among IgAN patients (5). LPS, a classical endotoxin derived from the outer membrane of Gram-negative bacteria, serves as an indispensable virulence factor that triggers Toll-like receptor-4 (TLR-4) inflammatory signaling, eliciting potent innate and adaptive immune responses (22). Therefore, we designated the established IgAN model with and without systemic administration of LPS as “immune activation (IA)/immune remission (IR)” and “immune stability (IS)”, respectively (Methods). Accordingly, we generated temporal scRNAseq data and scATACseq data for CD45-positive immune cells isolated from kidneys in different immune responses to delineate population heterogeneity and identify gene expression as well as accessible chromatin patterns associated with major branches of immune cell phenotype. To date, this study represents the first multiomics analysis that characterizes the dynamic immune cellular landscape at a high resolution in an early IgAN mouse model.

## 2 Results

### 2.1 A murine model with LPS injection to phenocopy 3 states in early human IgAN-associated glomerulopathy

**Figure 1:**
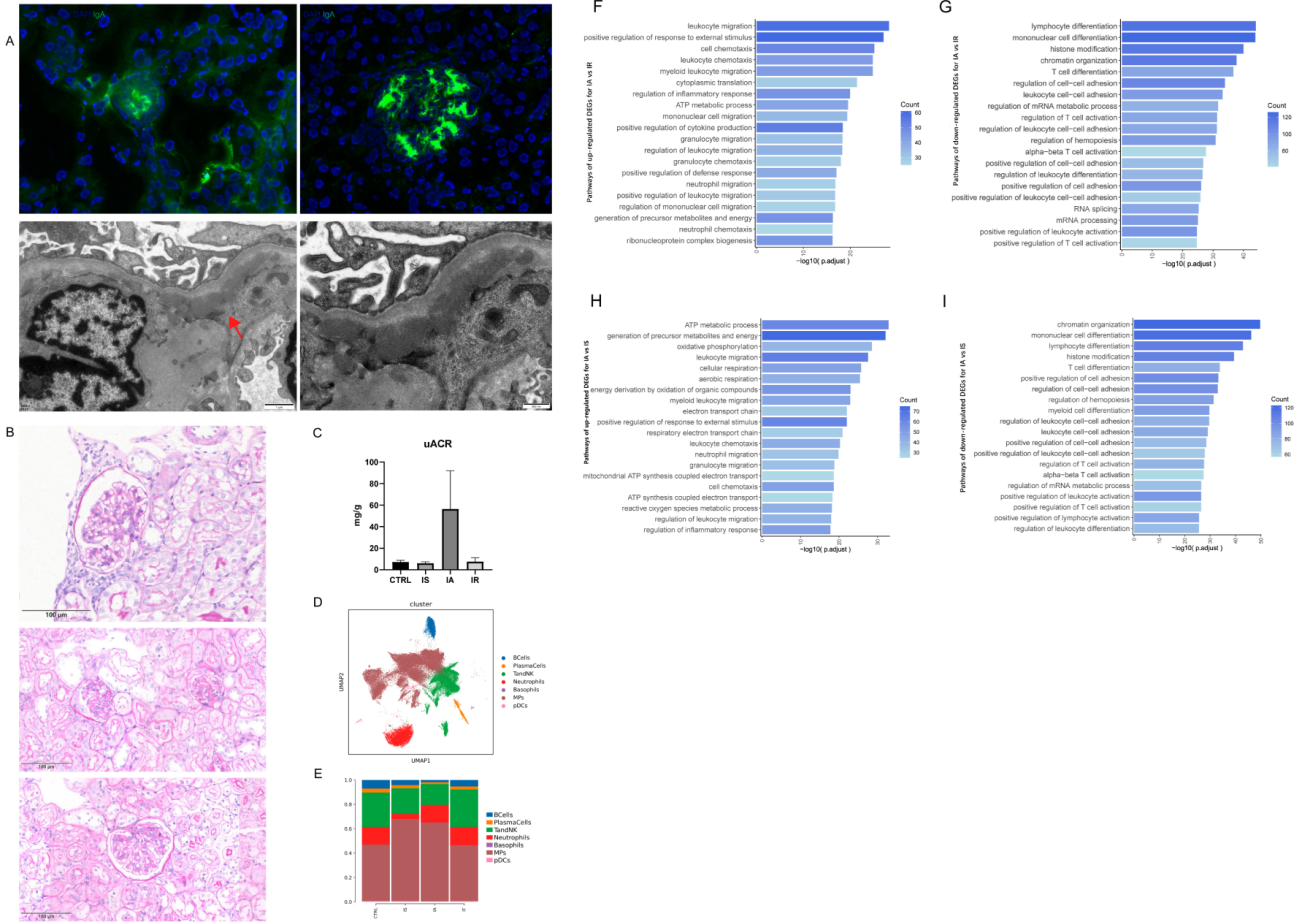
Single cell transcriptional immune landscape of acute exacerbation in an early IgAN model. **(A)** Representative analysis of IgA glomerular deposits in IgAN groups (upper left) and CTRL groups (upper right) using immunofluorescence. Representative analysis of IgA glomerular deposits (red arrow) in IgAN groups using transmission electron microscope (bottom). **(B)** Representative histologic images of periodic acid-Schiff (PAS) staining across IgAN groups(upper: IA, middle: IR, bottom: IS). Mild increased cellularity of mesangial cells can be widely observed, while immune cell infiltration, adhesion of Bowman’s capsule, and renal tubule atrophy can be detected in some samples. **(C)** Urinary albumin-to-creatinine ratios (UACRs) in CTRL, IS, IA and IR. **(D)** UMAP showing the distribution of CD45^+^ cells from the scRNAseq data. Each point represents one cell. The cells are marked by color code based on the cell type. **(E)** Histogram depicting the proportion of CD45^+^ cells per cell type. **(F)** Barplots showing enriched pathways of upregulated DEGs for IAvsIR. **(G)** Barplots showing enriched pathways of down-regulated DEGs for IAvsIR. **(H)** Barplots showing enriched pathways of up-regulated DEGs for IAvsIS. **(I)** Barplots showing enriched pathways of down-regulated DEGs for IAvsIS.

Established IgAN model in this study showed prominent glomerular immune deposits (**Figure 1A**) with histopathologic changes (**Figure 1B**) as observed in early IgAN patients. Despite the LPS injection in the IA or IR group not inducing more severe detectable glomerular abnormalities, physiological measurements confirmed that the IA group (short for IA) exhibited more severe proteinuria, compared to the other groups (**Figure 1C**). Therefore, these three phases simulate to a certain extent the onset and resolution of self-limited AKI in early IgAN.

### 2.2 CD45^+^ cell transcriptional atlas in mouse kidneys

Dissociated kidney cells from CTRL, IS, IA and IR (4 or 5 mice each) were processed and enriched for CD45-positive immune cells (Methods and **Supplementary Figure S1**). Isolated CD45^+^ cells were then pooled into a single sample per experimental condition for scRNAseq and scATACseq analysis respectively using the 10× Genomics platform. After quality control and filtering (Methods and **Supplementary Figure S2**), we obtained 72304 CD45^+^ single-cell transcriptomes from vehicle and IgAN mice.

Seven major cell types were identified on the basis of the canonical marker expressions as B cells, plasma cells, T and NK cells (TandNK), neutrophils, basophils, mononuclear phagocytes (MPs), and plasmacytoid dendritic cells (pDCs) (Methods and **Figure 1D**). MPs could be further divided into macrophages, monocytes, conventional type 1 dendritic cells (cDC1), and conventional type 2 dendritic cells (cDC2) (**Supplementary Figure S4**). The proportion of immune cell types showed difference in disease phases, and we detected a prominent dynamic change of the myeloid cells and the lymphoid cells (**Figure 1E**).

To investigate the distinctive immune profiles of different disease phases, we performed Gene Ontology (GO) pathway analysis of DEGs for IA, IR and IS. We found increased expression of genes that regulate leukocyte migration and chemotaxis, especially mononuclear cells and granulocytes in IA compared to IR (**Figure 1F**), indicating that circulatory myeloid activation altered the kidney myeloid compartments during the early onset of AKI. While in IR and IS, DEGs were more enriched in leukocyte differentiation, cell-cell adhesion and T cell activation than in IA (**Figures 1G, I**). When compared with IS, IA was characterized by elevated levels of cellular metabolism, including generation of metabolites and cellular respiration (**Figure 1H**), consistent with the activated state of immune cells.

### 2.3 Temporal scRNAseq reveals transcriptional heterogeneity in macrophage subsets

**Figure 2:**
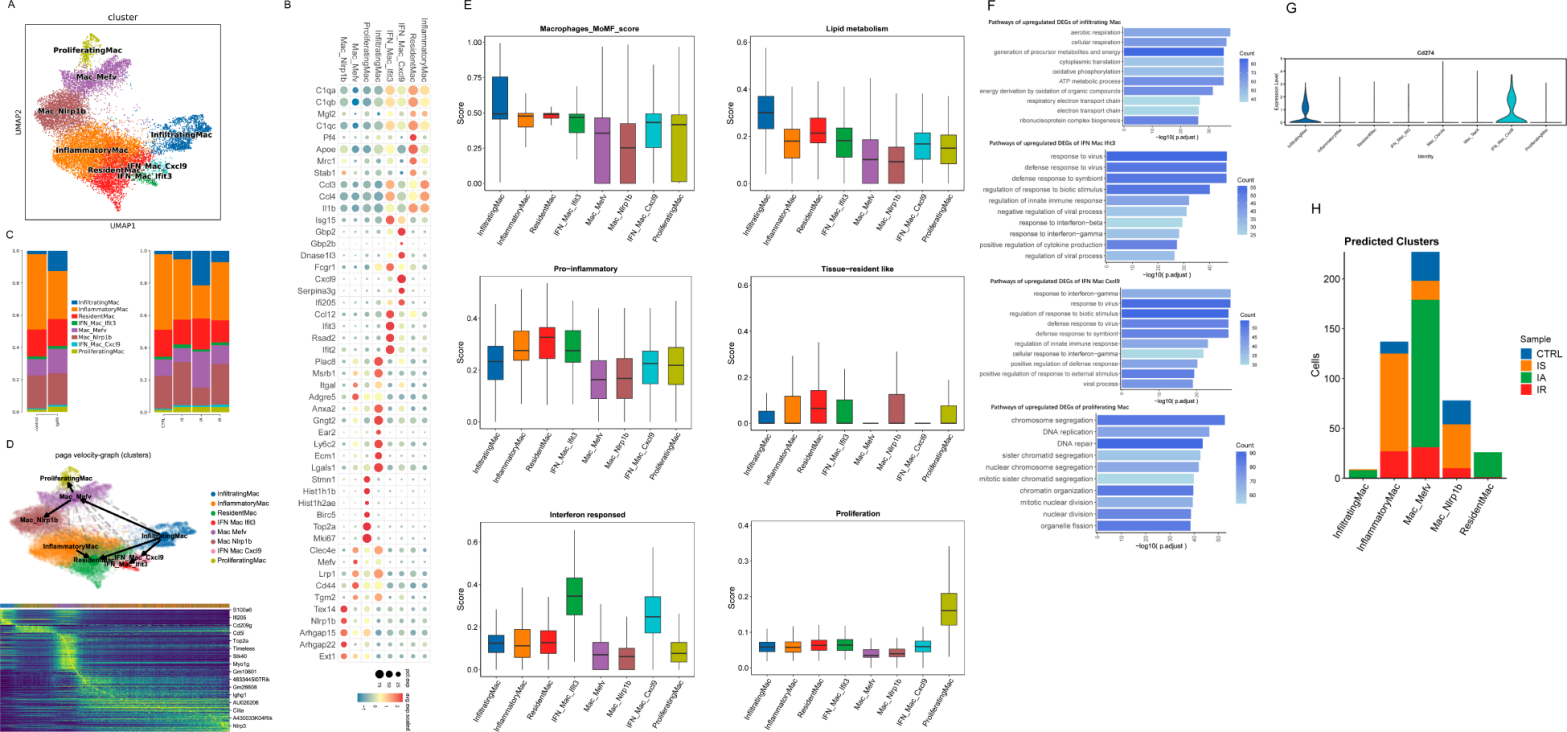
Immunological features of macrophage subsets. **(A)** UMAP of macrophages from scRNA-seq colored by cell type. **(B)** Dot plot of selected average gene expression values (log scale) and percentage of macrophages expressing these genes within each cluster. **(C)** Histogram depicting the proportion of macrophages per cluster. **(D)** PAGA pseudo-time network and embedding celltype showing the transition trajectories among macrophage subsets. Heatmap of genes that are differentially expressed along the trajectories elicited by RNA velocity. **(E)** Boxplot showing the indicated functional scores of macrophage subsets. **(F)** Barplots showing enriched pathways of upregulated DEGs of indicated macrophage subset. **(G)** Violinplot showing the expression of Cd274 in macrophage subsets. **(H)** Predicted cluster identity of proliferating cells shown as the number of cells per cluster and colored by sample.

To examine the kidney macrophage heterogeneity, we performed the unsupervised clustering of macrophages from vehicle and IgAN immune cells, which identified 8 subclusters, including infiltrating macrophages (infiltrating Mac, highly expressing infiltrating macrophages genes e.g., *Plac8*, *Msrb1*, *Anxa2*, *Gngt2*, *Ear2*), resident Mac, inflammatory Mac (highly expressing inflammatory macrophages genes e.g., *Ccl3*, *Ccl4*, *Il1b*), 2 subsets of interferon (IFN) gene signature high Mac (IFN Mac), proliferating Mac (highly expressing proliferating macrophages genes e.g., *Stmn1*, *Hist1h1b*, *Hist1h2ae*, *Birc5*, and *Mki67*), NLR family, pyrin domain containing 1B (*Nlrp1b*)-high expressing Mac (Nlrp1b Mac) and Mediterranean fever (*Mefv*) -high expressing Mac (Mefv Mac) (**Figures 2A, B**) (23). The annotations were identified based on previously defined marker genes by Fu et al. (23). The cell types in the current study were largely similar with that in the mouse model of early diabetic kidney disease (DKD), implicating that IgAN and DKD might share conserved phenotypic spectrum in local macrophage transcriptome, despite of different mouse model from different background (23). Mannose receptor C-type 1 (Mrc1)-high expressing (Mrc1^hi^) Mac, one of resident macrophage subsets as Fu et al. have reported, showed relatively higher expression of resident marker genes (e.g., *C1q*, *Cd81* and *Mgl2*) than other subclusters in our study, thus we annotated them as resident Mac (**Figure 2B**). Although we did not detect any populations with a relatively high expression of *Trem2* (**Supplementary Figure S4**), we distinguished 2 subsets Nlrp1b Mac and Mefv Mac that have not been previously described and might be specific to IgAN. And we observed notable changes in the proportions of Nlrp1b Mac and Mefv Mac in IA (**Figure 2C**), suggesting their potential role in the acute phase of IgAN or kidney injury. Besides, we split IFN Mac into 2 subsets as it clustered “apart” in the scRNAseq UMAP space and they displayed different IFN-stimulated gene (ISG) expression patterns that IFN Mac Cxcl9 with a high expression of *Gbp2*, *Gbp2b* and *Cxcl9*, while IFN Mac Ifit3 with a high expression of *Isg15*, *Ccl12*, *Ifit3* and *Ifit2* (**Figures 2A, B**).

We first examined the macrophage subsets for the expression of canonical M1 markers and M2 markers, which showed an increasing trend for both M1- and M2-like macrophage subtypes, rather than having discrete M1 or M2 phenotypes (**Supplementary Figure S5**). Recent evidence from scRNAseq analysis has pointed to a more dynamic and continuous spectrum of macrophage polarization phenotypes of tissue macrophages under various conditions (23, 24).

To understand the potential developmental transitions of macrophage clusters, we applied RNA velocity analysis to construct the developmental trajectories of 8 macrophage clusters. Two major trajectories were observed that started from infiltrating Mac, then bifurcated and ended up as Nlrp1b Mac and resident Mac respectively, demonstrating that there might be a constant flow of infiltrated macrophages entering these clusters and terminally differentiating as them (**Figure 2D**). In line with previous publications, resident macrophages or other subclusters are both locally proliferating and partially replenished by the circulation especially when space in the resident macrophage pool is created under inflammatory conditions (24, 25). **Figure 2D** shows examples of genes that are highly expressed along the trajectories (e.g., pro-inflammatory gene *S100a6* and ISG *ifi205* in infiltrating Mac, complement activation gene *Cd5l* and phagocytosis-associated gene *Myo1g* in Mefv Mac).

By examining signature genes defined by prior data (Methods and **Supplementary Table S1**), we observed distinct functional status for each macrophage subset as follows: infiltrating Mac with the highest lipid metabolism score and a high monocyte-derived macrophage (MoMF) score (**Figure 2E**). And GO analysis revealed that many pathways specifically enriched in infiltrating Mac were related to energy metabolic process and respiration (**Figure 2F**). Macrophages augmenting lipogenesis and the utilization of glucose upon the stimulation of TLR4 by LPS has been linked to enhanced phagocytosis and cytokine production (26–28). We also observed high pro-inflammatory scores in resident Mac, inflammatory Mac and IFN Mac Ifit3, the highest resident score for resident Mac, as well as the highest interferon-responsed score for IFN Mac (**Figure 2E**). Consistent with its pro-inflammatory potential, IFN Mac Ifit3 DEGs were enriched in pathways related to defense response to virus and cytokine production, whereas IFN Mac Cxcl9 in pathways associated with nucleotide metabolic process (**Figure 2F**). Next, we asked whether IFN Mac Cxcl9 had anti-inflammatory potential by examining the *Cd274* (encoding PD-L1) expression of macrophages. IFN Mac Cxcl9 was found to show a marked expression (**Figure 2G**). Together, these results further confirmed the bifurcation of IFN Mac. Of interest, research has shown that tumor T cells cocultured with the PD-L1^+^ macrophages exhibited an impaired production of IFN-γ (29). In other words, IFN Mac Cxcl9 might inhibit T cell activity and suppress the overexpression of IFN. Furthermore, IFN Mac Cxcl9 displayed a relatively high score of immune regulation and high expression of immuneregulatory gene *Sod1* (**Figure 2E**; **Supplementary Figure S5**). Altogether, these observations indicated a key role for this subset in the ability to attenuate and resolve the inflammation. There was also a proliferating cluster characterized by the expression of genes consistent with DNA replication and repair, as well as cell division, such as *Hist1h1b*, *Hist1h2ae*, *Birc5*, and *Mki67* (**Figures 2B, F**). Because cell cycle genes can obscure underlying transcriptional identity, we projected these cells back onto the remaining clusters as described in previous publications (Methods) (20). Most proliferating cells belonged to the IA Mefv cluster, with part of cells derived from inflammatory Mac and Nlrp1b Mac present at IS (**Figure 2H**). Thus, these clusters shared transcriptional features of proliferative activity that might drive colocalization in scRNAseq space.

GO pathway analysis of Nlrp1b Mac DEGs disclosed their various regulatory effects on immune response and apoptotic signaling pathway, together with the upregulation of genes linked to leukocyte migration, chemotaxis and cell-cell adhesion, whereas Mefv Mac DEGs were positively correlated with the regulation of leukocyte differentiation and GTPase activity (**Supplementary Figure S6**). However, these “regulatory-alike” clusters did not belong to the canonical immunoregulatory cells due to their low immune-regulatory score as shown in **Supplementary Figure S6**. They did not acquire anti-inflammatory functions that contributed to tissue fibrosis, angiogenesis or inhibition of T cell responses either (**Supplementary Figure S6**). Further looking at other immunoregulatory genes defined by Zhang et al. revealed a significant upregulation of *Lyn*, *Mtss1* and *Pecam1* (30). These analyses unveiled a previously unappreciated macrophage population with potent immunoregulatory effects.

To gain more molecular insights into these clusters, next we used gene activity, a metric of local gene accessibility, to approximate gene expression and avoid the interferences caused by overexpression of mitochondrial genes or ribosomal genes. First we compared Nlrp1b Mac and Mefv Mac to resident Mac, the representative populations of the two trajectories. Nlrp1b Mac and Mefv Mac were distinguished by another immunoregulatory gene *Ptprd*, genes associated with cell adhesion (*Nrxn3*, *Tenm2* and *Grid2*) and transcription factor (TF) ESRRG (**Supplementary Figure S7**). Of note, the largest number of genes with differential activity found in this comparison were down-regulated genes of Nlrp1b Mac and Mefv Mac, including *Rab7b* (expression involved in response to IFN), *Cd52* (expression involved in response to bacterium), *Cd83* (expression involved in positive regulation of CD4^+^ T cell differentiation), *C1qc* and *H2-Eb1* (expression involved in phagocytosis and antigen presentation), and migration-related genes *Ccl3* and *Ccl4* (**Supplementary Figure S7**). Then based on comparison with each other, Nlrp1b Mac had higher expression of *Ptprd*, whereas more immunoregulatory genes *Tnfrsf1b* and *Pik3ap1* were higher in Mefv Mac (**Supplementary Figure S7**). These data further confirmed the distinct immunoregulatory effects of Nlrp1b Mac and Mefv Mac.

In summary, these non-canonical immunoregulatory cells with high expression of multiple related genes and various biological effects did not demonstrate a typical pro- or anti-inflammatory phenotype. Next, we performed ligand-receptor (L-R) analysis to explore molecular crosstalks between these cells and the rest macrophages or CD4^+^ T cells. We found that APP-CD74 showed the highest interaction potential (**Supplementary Figure S8**). Amyloid beta precursor protein, encoded by the *App* gene, is involved in negative regulation of blood circulation; positive regulation of endothelin production; and positive regulation of tumor necrosis factor (TNF) production, and CD74 is mainly involved in macrophage migration inhibitory factor signaling pathway. In addition, it has been recently reported that the APP-CD74 axis contributes to Treg-exhaustion CD8^+^ T cells (Tex) interaction in HBV-infected patients (30). Together, these data may provide valuable information for functional verification of these immunoregulatory cells in future studies regarding immune pathogenesis as well as therapeutic attempts for IgAN.

### 2.4 Dynamic variances in neutrophil profile across different phases

**Figure 3:**
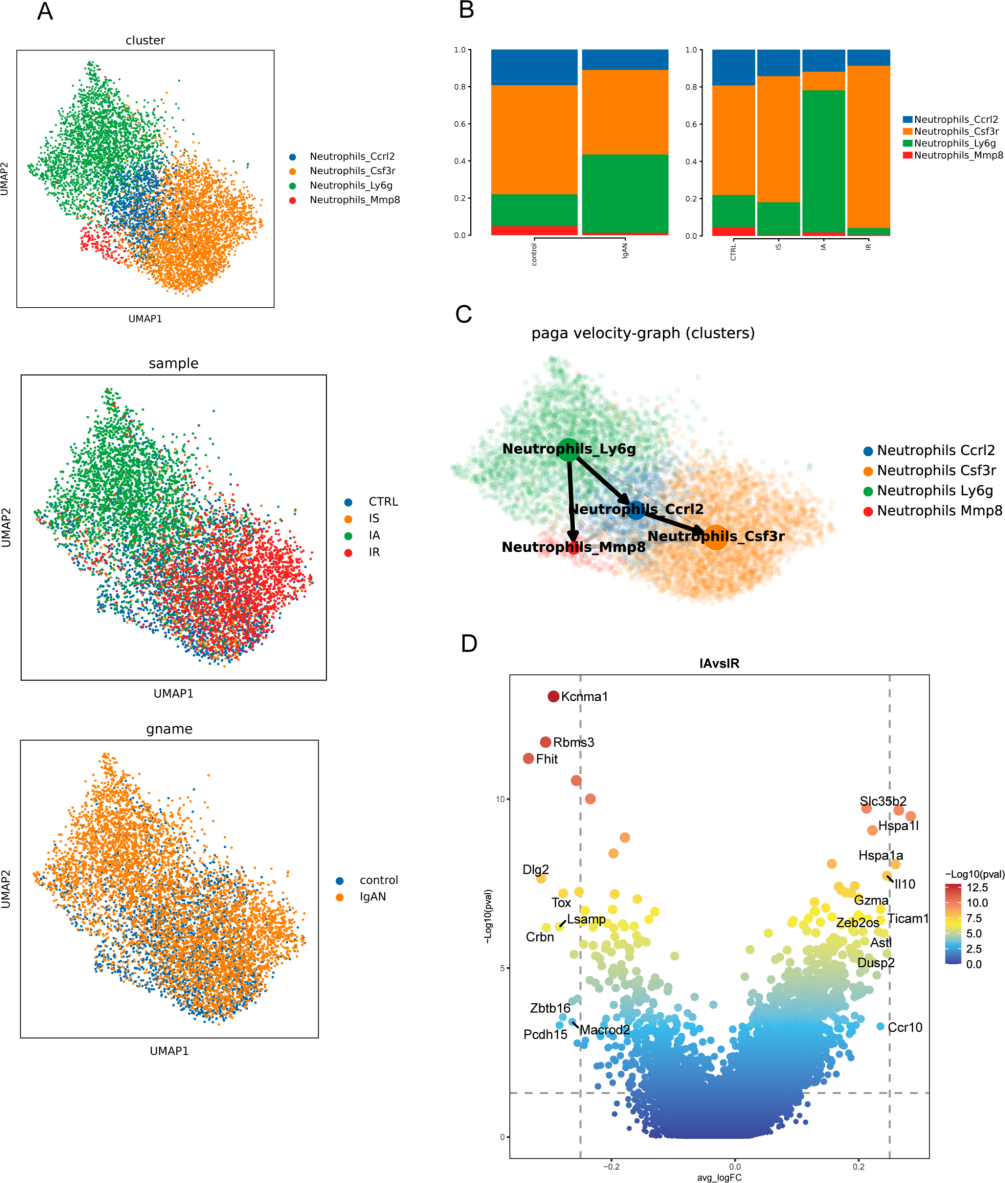
Immunological features of neutrophil subsets. **(A)** UMAP of neutrophils from scRNA-seq colored by cell type (upper), group (middle) and sample (bottom). **(B)** PAGA pseudo-time network and embedding celltype showing the transition trajectories among neutrophil subsets. **(C)** Bar graph showing proportions of neutrophil subsets in different groups and samples. **(D)** Volcano plots showing differential gene activity between neutrophils from IA and IR.

Neutrophils were further divided into Ccrl2, Csf3r, Ly6g and Mmp8 subclusters based on their high expression of the marker gene *Ccrl2*, *Csf3r*, *Ly6g* and *Mmp8* respectively (**Figure 3A**). **Supplementary Figures S9, S10** show enrichment scores of neutrophil subsets for specific gene signatures and GO pathways that are specifically enriched in each neutrophil subset. Ccrl2 neutrophils were characterized by high pro-inflammatory score and involved in multiple signaling pathways such as NF-κB signaling, pattern recognition receptor (PRR) signaling pathway, and extrinsic apoptotic signaling pathway (**Supplementary Figures S9, S10**). Ly6g neutrophils with a high ISG score and the highest pre-neutrophil score (**Supplementary Figure S9**), serving as the starting root of the pseudo-time trajectories (**Figure 3B**), exhibited characteristics indicative of both early differentiated and viral response. Mmp8 neutrophils were distinguished by chemotaxis and migration-related genes and had more pro-angiogenic potential (**Supplementary Figures S9, S10**).

The proportion of neutrophil cell types was largely similar between CTRL and IS (**Figure 3C**). However, in IA there was skewing away from Csf3r neutrophils and toward the Ly6g subset (**Figure 3C**). While in IR, there was a substantial increase in Csf3r neutrophils with concomitant loss of Ly6g subset (**Figure 3C**), suggesting the specific neutrophil response might contribute to control the acute exacerbation of IgAN. Based on gene activity, neutrophils in IA highly expressed *Il10*, *Adora2a*, *Tnip1*, *Pou2f2*, *Clec4a1*, *Lta*, etc., genes mainly involved in positive regulation of inflammatory response through multiple signaling pathway (**Figure 3D**), including MYD88-dependent TLR signaling pathway, NF-κB signaling, G protein-coupled receptor signaling pathway, IL17-mediated signaling pathway, type I IFN-mediated signaling pathway. While looking at neutrophils from IR, representative genes *Il1r2*, *Il15*, *Zfp36l2* (negative regulation of cell differentiation) and *Wbp1l* (CXCR4 signaling pathway) were relatively up-regulated (**Figure 3D**). Taken together, these findings highlighted the potential roles of neutrophils in the pathogenesis of IgAN that have rarely been explored.

### 2.5 The integrated immune landscape distinguishes Nlrp1b Mac as a distinct macrophage subset

**Figure 4:**
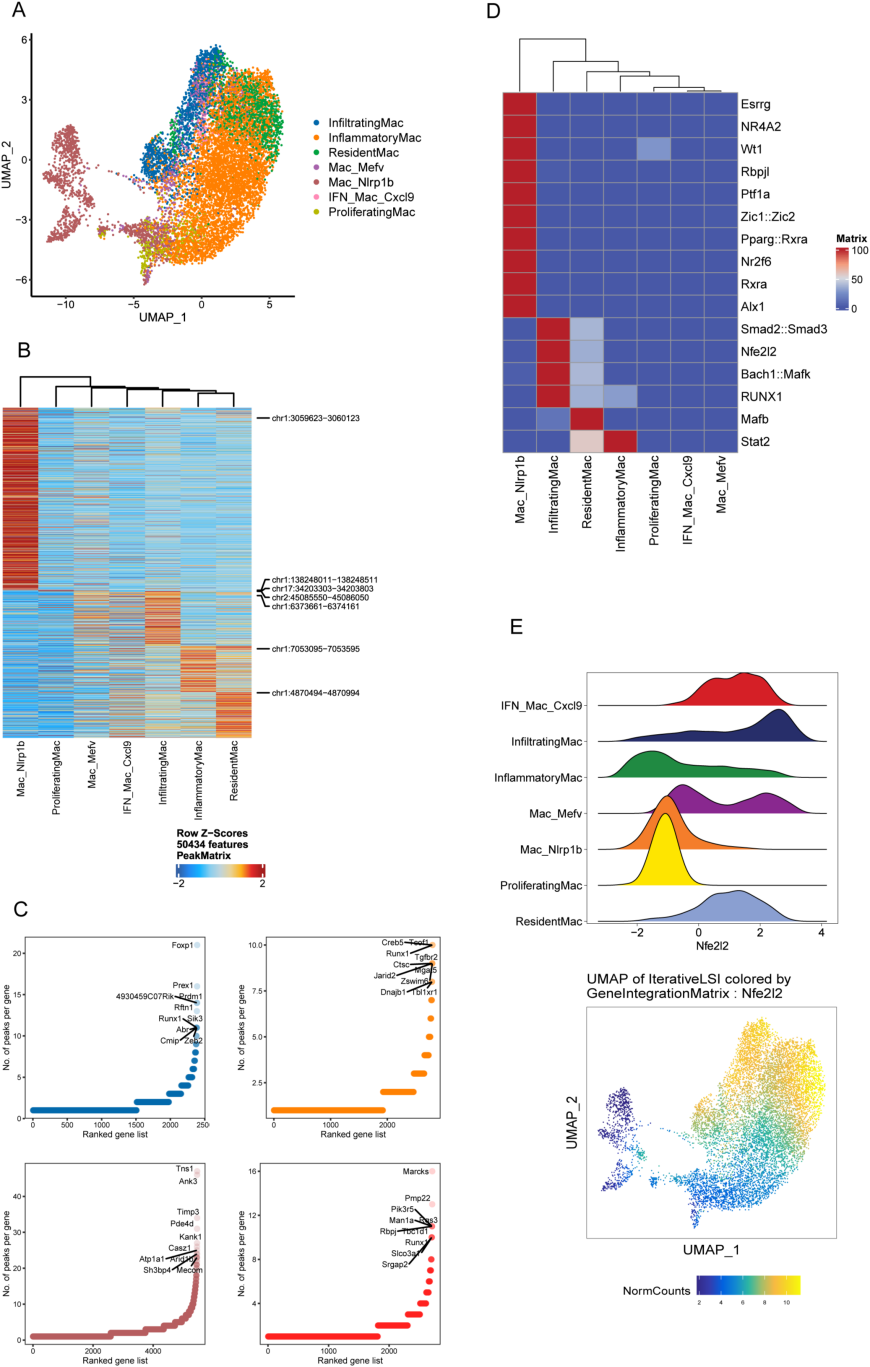
The integrated immune landscape distinguishes Nlrp1b Mac as a distinct macrophage subset. **(A)** UMAP showing the distribution of macrophages using the scATACseq and scRNAseq data in the LSI space. Each point represents one cell. The cells are marked by color code based on the different clusters. **(B)** Average accessibility of DACRs per scATAC-seq macrophage cluster. **(C)** Number of DACRs per gene loci for 4 representative scATAC-seq cluster: infiltrating Mac (blue), inflammatory Mac (yellow), resident Mac (red) and Nlrp1b Mac (brown). **(D)** Heatmap of TF motif deviation scores of macrophage subsets. **(E)** Ridge plot showing NFE2L2 deviation scores of macrophage subsets (upper) and UMAP showing the distribution of NFE2L2 using the scATACseq and scRNAseq data in the LSI space (bottom).

Distinct cell type is the result of an epigenetically distinct developmental path driven in part by specific TF (31). Next, we proceeded to explore the epigenetic and transcriptomic landscape of immune cells to identify the regulatory elements that define the biological states among the four groups based on parallel scRNAseq and scATACseq on the same samples. As shown in the UMAP, following the integration of scATAC- and scRNA-seq datasets, 2 major clusters and 7 celltypes were identified (**Figure 4A**; **Supplementary Figure S11**), validating that scATACseq reveals fewer cell “fates” underlying multiple transcriptional states (20). Unexpectedly, Nlrp1b Mac stood out as a distinct population outside of other clusters (**Figure 4A**). Given the epigenetic divergence of Nlrp1b Mac versus other macrophage clusters, we next compared chromatin accessibility changes between them. Among regions with increased or lost accessibility, only a finite number of them were shared (**Supplementary Figure S11**).

Next, we investigated epigenetic programs used by different macrophage subsets as described by Giles previously (20). We visualized all differentially accessible chromatin regions (DACRs) (**Figure 4B**), then assessed the number of DACRs in each gene locus (**Figure 4C**). This approach revealed four representative global patterns of distinct ACRs among macrophage clusters, namely Nlrp1b Mac, infiltrating Mac, inflammatory Mac and resident Mac (**Figures 4B, C**). Accordingly, Nlrp1b Mac had a unique ACR profile and had the most accessible DACRs among macrophage clusters (**Figures 4B, C**).

Nlrp1b Mac have a unique TF motif profile characterized by enrichment in ESRRG, NR4A2, RBPJL, WT1, ZIC1::ZIC2, etc. (**Figure 4D**; **Supplementary Figure S12**). In this case, we observed a specific TF regulation activity pattern in these clusters with low deviation score of NFE2L2 in Nlrp1b Mac and proliferating Mac, and high score in IFN Mac Cxcl9, infiltrating Mac and resident Mac (**Figure 4E**). Since most DEGs of Nlrp1b Mac were shared with other Mac (**Supplementary Figure S13**), these underscore the potential of chromatin accessibility to provide additional information beyond transcriptional data (20).

### 2.6 Tex/CX3CR1^-^transTeff is the major CD8+ effector during the acute phase

**Figure 5:**
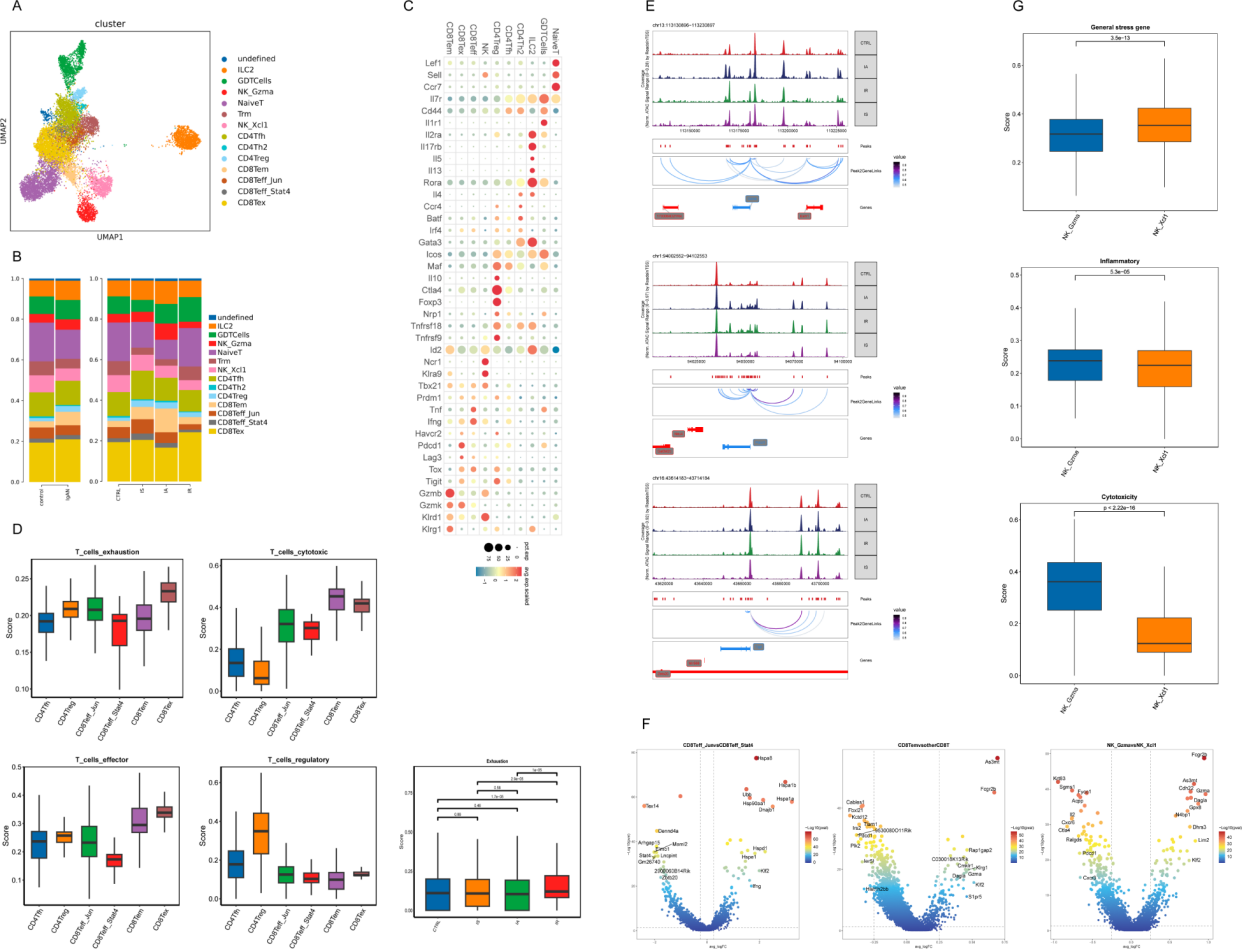
Immunological features of CD8^+^ T-cell and NK-cell subsets. **(A)** UMAP of T and NK cells from scRNAseq colored by cell type. **(B)** Histogram depicting the proportion of cells per cluster. **(C)** Dot plot of selected average gene expression values (log scale) and percentage of cells expressing these genes within each cluster. **(D)** Boxplot showing the indicated functional scores of T-cell subsets and exhaustion score of Tex across samples generated on integrated scRNAseq data. **(E)** Genome accessibility tracks of indicated gene loci with peak-to-gene links identified by ArchR across samples. The genes are depicted in red when located on the positive strand (TSS on the left) and in blue when situated on the negative strand (TSS on the right). The grey boxes highlighted the enhancer or promoter regions of the gene of interest. **(F)** Volcano plot showing DEGs between Teff_Jun and Teff_Stat4, differential gene activity between Tem and other CD8^+^ T cells, and differential gene activity between NK_Gzma and NK_Xcl. **(G)** Boxplot showing the indicated functional scores of NK-cell subsets generated on integrated data.

Further clustering of scRNAseq T and NK cells yielded 12 clusters, mainly including type 2 innate lymphoid cells (ILC2), γδ T cells (GDT), naïve T cells, CD4^+^ T-follicular helper cells (Tfh), regulatory CD4^+^ T cells (Treg), effector memory CD8^+^ T cells (Tem), 2 effector CD8^+^ T cells (Teff_Jun and Teff_Stat4 with their high expression of the marker gene *Jun* and *Stat4* respectively) and exhaustion CD8^+^ T cells (Tex) (**Figures 5A-C**). These clusters were similar to those resolved and annotated from the integrated data (**Supplementary Figure S14**). This further validated the accuracy of the cell type annotation, and demonstrated that unlike the Mac subpopulation, which has a common lineage “fate”, the heterogeneity of T cell subpopulations is relatively stable and conserved from both transcriptomic and epigenetic perspective. Therefore, subsequent analyses are mostly based on the integrated scRNAseq and scATACseq data to reduce potential errors and improve the reliability of the results.

Although Tex herein displayed a typical exhausted state, which was featured by high expression of inhibitory markers (*Tox*, *Havcr2*, *Lag3*, *Pdcd1* and *Tigit*) and low expression of effector markers (*Tbx21* and *Ifng*), their high expression of *Gzmk* and intermediate expression of *Lag3* among other genes could term them in a different way as predysfunctional cells identified in a melanoma cohort (32) (**Figure 5C**). Distinguished from the “authentic” progenitor Tex or Tex precursor (Tpex) with naïve-associated genes (*Lef1*, *Sell*, *Ccr7* and *Il7r*) expression, Tex or predysfunctional cells herein with expression of Teff genes *Tbx21* and *Zeb2* more resemble transitory Teff (transTeff) (Tpex→transTeff→termTex), the latter were previously described as Tpex-derived effector-like CX3CR1^+^ T cells during mouse lymphocytic choriomeningitis virus (LCMV) infection (20, 33, 34) (**Figure 5C**; **Supplementary Figure S15**). Despite transTeff in our study with loss of CX3CR1 (**Supplementary Figure S15**), their effector pattern was defined by high cytotoxic score and effector score as shown in **Figure 5D**. Given the observed increase in the proportion and exhaustion score of Tex in IR (**Figures 5B, D**), we have deduced that CX3CR1^-^transTeff represents the predominant CD8^+^ effector population during IA, which subsequently evolves towards termTex from IA to IR.

We next asked how key changes in chromatin accessibility identified by scATACseq were associated with developmental trajectories. We identified distinct epigenetic patterns associated with expression of key genes (*Gzmk*, *Pdcd1* and *Tigit*) across time points using scATACseq (**Figure 5E**). The increased accessibility of the *Gzmk* locus in IA and the *Pdcd1* locus in IR was observed as anticipated (**Figure 5E**). Interestingly, we observed increased accessibility at the promoter and enhancers regions of *Gzmk* in IS (**Figure 5E**).

Lastly, we noted a unique feature of heat-shock protein (HSP) genes (*Hspa8*, *Hspa1b*, *Hspa1a*, *Dnajb1*, *Hspd1*, *Hspe1*) overexpression in Teff_Jun (**Figure 5F**; **Supplementary Figure S14**). A CD8^+^ T cell population with HSP genes upregulated was previously reported in Tex isolated from the mouse spleen during LCMV infection, but their biological function remained unelucidated (20). Further studies are needed to clarify the significance of Teff_Jun in IgAN.

Similarly, to observe distinct functional status for each T-cell and NK-cell subset, we examined signature genes defined previously on the integrated scRNAseq data (Methods and **Supplementary Table S1**). As expected, the highest regulatory score for Treg and the highest exhaustion score for Tex confirmed their transcriptomic signature (**Figure 5D**). We also observed the highest cytotoxic score for Tem (**Figure 5D**). As for NK subsets, NK_Gzma (with high expression of marker gene *Gzma*) had a significantly higher cytotoxic score, while NK_Xcl (with high expression of marker gene *Xcl*) had a relatively higher expression of general stress-related genes, a pattern reminiscent of CD8^+^ Tem and Teff_Jun (**Figure 5G**; **Supplementary Figure S14**). Tem and NK_Gzma might belong to one epigenetic group that featured by accessibility at *As3mt*, *Dagla*, NK-associated gene *Fcgr2b* and cytotoxic gene *Gzma* (**Figure 5F**). Similar to recent work, our analyses also identified specific T-cell cluster expressing genes associated with NK cells (*Klr* genes or *Fcgr2b*, for example) (20, 35, 36) (**Supplementary Figure S1**).

### 2.7 Cell-cell interaction between immune cells and endothelial cells

**Figure 6:**
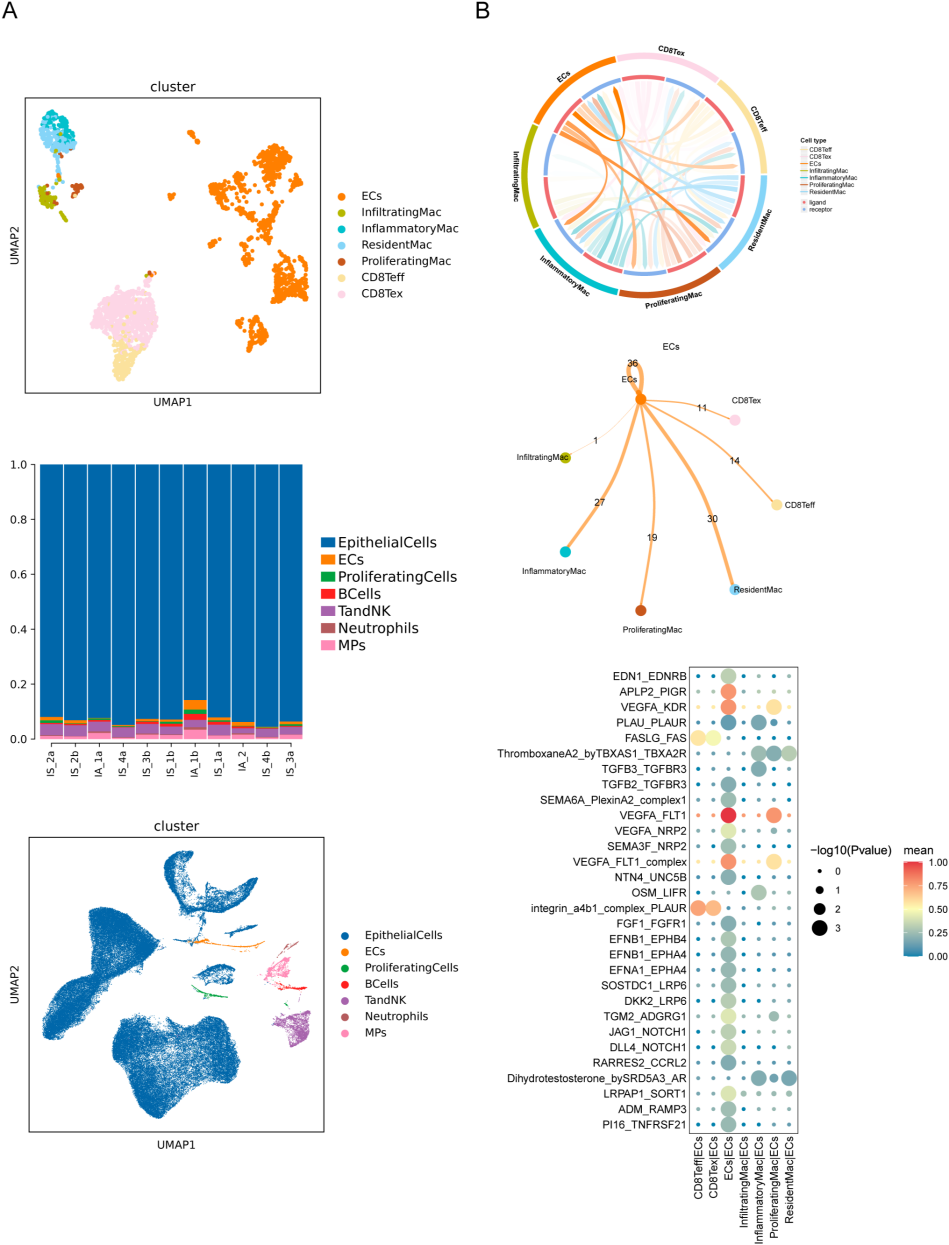
Cell-cell interaction between immune cells and endothelial cells. **(A)** UMAP of indicated clusters from scRNAseq colored by cell type and proportion of cells per cluster. **(B)** Visualized network graphs showing the cell-type-specific cell-cell interactions. Cell-cell communication network showing quantified signaling strength from ECs to all cell types. The counts highlight the numbers of L-R pairs between two cell types. Bubble heatmap showing the potential cytokine-related, grow factor-related and top 30 L-R pairs between indicated cells.

To dissect the potential crosstalk of immune cells and renal resident cells, we performed scRNAseq on kidney cells obtained from an independent cohort of IS and IA mice without immune-cell isolation (**Figure 6A**). Several clusters or subsets were undetected because of their relatively low overall proportions (**Figure 6A**). Then we used Cellphone DB to infer cell–cell interaction between macrophage or CD8^+^ T-cell subsets and endothelial cells (ECs), considering prior findings that ECs have a key role in the activation/recruitment of leukocytes at the initial stages of IgAN (37) (**Figure 6**). We detected a wide range of interaction events among ECs, Teff, Tex, resident Mac, inflammatory Mac and proliferating Mac, and the most prevalent ones occurred in ECs, resident Mac and inflammatory Mac (**Figure 6B**). By examining L-R pair interactions, several L-R pairs between vascular endothelial growth factor A (VEGFA) and its receptors (KDR and FLT1) exhibited strong potential interaction among ECs themselves and between ECs and proliferating Mac (**Figure 6B**). This growth factor induces proliferation of vascular ECs and angiogenesis. While FASLG-FAS and integrin a4b1 complex-PLAUR showed higher interaction potentials between CD8^+^ T-cell subsets and ECs (**Figure 6B**), which are involved in the apoptotic signaling pathway. These results highlighted the different roles for macrophages and CD8^+^ T cells in maintaining homeostasis of ECs under stress.

## 3 Discussion

In our study, an IgA nephropathy (IgAN) model with an extra injection of LPS was established to imitate the onset and resolution of self-limited AKI typically seen in early stages of IgAN. Through longitudinal use of scATAC- and scRNA-seq, we generated a comprehensive cellular landscape of renal immune microenvironment. We obtained a total of 72,304 single-cell transcriptomes from CTRL, IS, IA and IR mice, enabling us to perform a high-resolution mapping of all major immune cell types with an additional detailed analysis of macrophage, neutrophil, CD8^+^ T-cell and NK-cell subsets.

Our data provide an unbiased illustration of the immunological hallmarks as the proportions of immune cell types varied during disease phases. IS is largely similar to the indolent clinical course seen in most IgAN patients, exhibiting low-grade inflammation in the kidney. Although there was a greater shift in the proportion of MPs in comparison to the control kidneys, we nevertheless detected subtle alterations in the proportion of cell subtypes of MPs (**Figure 1E**). In IA, the initial phase of AKI, there was an increased proportion of infiltrating Mac, Mefv Mac, Ly6g neutrophils, NK_Gzma and Tem, together with a relatively decreased proportion of inflammatory Mac, Nlrp1b Mac, Csf3r Neutrophils and Tex (**Figures 2B**, **3B**, **5B**). While in the later stage, IR was characterized by an increased proportion of Csf3r Neutrophils and Tex, along with a relatively decreased proportion of Ly6g neutrophils, contrary to IA (**Figures 3B**, **5B**). These changes collectively contribute to their distinct immune profiles. As neutrophils was the ones with the most dramatic alteration among these cell types, our findings underscore the potential significance of neutrophils in the pathogenesis of IgAN, which has been rarely explored.

These analyses also uncovered new subpopulations, including an epigenetically distinct macrophage subset with potential immunoregulatory effects (Nlrp1b Mac), and an early subset of Tex distinguished by effector and cytolytic potential (CX3CR1^-^transTeff). Our insights into these cell identities may help identify specific targets or pathways for future therapeutic manipulation. However, further investigation incorporating higher-dimensional profiles in the context of IgAN or kidney disease is imperative to resolve the different levels of dysfunctionality within the CD8^+^ T cell compartment and create a consensus nomenclature (38).

Lastly, our analysis detected a wide range of interaction events among endothelial cells, Teff, Tex, resident Mac, inflammatory Mac, and proliferating Mac, emphasizing the different roles for macrophages and CD8^+^ T cells in maintaining homeostasis of endothelial cells under stress. In conclusion, the study provides a comprehensive analysis of the dynamic changes in immune cell profiles in a model of IgAN, identifying key cell types and their roles and interactions. While these findings contribute to the understanding of IgAN pathogenesis and may provide potential targets for therapeutic intervention, it is important to acknowledge the limitations of the study. The injection of LPS alone cannot replicate the complex renal changes seen in various clinical AKI cases, therefore further validation of these results in human samples or *in vivo* is necessary once the experimental conditions are established.

## 4 Materials and methods

### 4.1 Mouse model

Male C57BL/6J mice, aged 11 ± 1 weeks and weighting 25 ± 2 g were obtained from SpePharm (Beijing) Biotechnology Co., Ltd. (Beijing, China) and maintained in a clean-grade room at controlled stable temperature and humidity. Our study exclusively examined male mice. It is unknown whether the findings are relevant for female mice. The IgAN mouse model was induced by “BSA + CCl4 + LPS” method as previously described (39). BSA (Sigma) dissolving water (200 mg/kg body weight) was gavaged every other day for 10 weeks, combined with subcutaneous injection weekly and intraperitoneal injection biweekly of CCl4 dissolved in castor oil (1:5; 0.1 ml). Then LPS (Sigma) (50 μg) was injected into tail vein at week six and eight. For the male weight-matched littermate controls served as the normal controls (designated as “CTRL” group), saline was used instead of the above reagents. The model was established at the end of the 10th week. IgA deposits in the glomeruli was observed by direct immunofluorescence, and transmission electron microscopy was also utilized to evaluate model establishment.

### 4.2 Experiment design

Twenty-four-hour urine samples were collected the day before renal tissues collected for subsequent experiments. For the IS group, IgAN mice were kept for at least 1 month without treatment after model establishment. For the IA and the IR groups, IgAN mice were subjected to tail intravenous injection of 1 mg/kg LPS the day and 5~7 days before samples and tissues collection, respectively (**Supplementary Figure S1**).

### 4.3 Kidney CD45-positive immune single-cell isolation and processing

Kidneys from 4 or 5 mice per experimental group (CTRL, IS, IA, IR) were processed together. Following exsanguination by perfusion of phosphate-buffered saline (PBS) containing 1 mM EDTA, mouse kidneys were cut into small pieces and digested on a rotor at 37 °C in Tissue Dissociation Mix (Singleron), according to the manufacturer’s instructions. Dissociated cell suspensions were filtered through a 40-μm cell strainer and further washed with calcium- and magnesium-free PBS. Leukocytes were enriched through 36%~72% Percoll (GE Healthcare) density gradient centrifugation before microbeads (Stem Cell Technologies) isolation, according to the manufacturer’s recommendations.

### 4.4 Histology and immunofluorescence

Fresh renal tissues were fixed in 4% paraformaldehyde and embedded in OCT separately. Four-μm thick formalin-fixed and paraffin-embedded sections were cut and subsequently deparaffinized for periodic acid-Schiff (PAS) staining. Images were acquired by PreciPoint M8 dual digital microscope & scanner. 10 μm frozen sections were made and slides were washed in 0.01M PBS for 20 min. Then the slides were further blocked with blocking buffer (5% bovine serum albumin with 0.1% Triton X-100) for 1 h. Fluorescein isothiocyanate (FITC)-labeled goat anti-mouse IgA (1:400, Abcam) was diluted by blocking buffer and incubated at 4°C overnight. The next day, slides were washed by 0.01M PBS for three times, 10 min each. Fluorescence images were acquired by Olympus VS120 Virtual Slide Microscope.

### 4.5 scRNAseq library generation

scRNAseq library construction was performed using the GEXSCOPE^®^ Single Cell RNA Library Kit Tissue V2 (Singleron Biotechnologies) as per the manufacturer’s protocol. Purified libraries were sequenced on an Illumina Hiseq X sequencer with 150 bp paired-end reads.

### 4.6 scATACseq library generation

scATACseq library construction was performed using the Nuclei Isolation for Single Cell ATAC Sequencing (10× Genomics; CG000169 Rev. E) and Chromium Next GEM Single Cell ATAC Reagent Kits v2 User Guide (10x Genomics; CG000496 Rev. A) as per the manufacturer’s protocol. Libraries were sequenced on an Illumina HiSeq X with 50 bp paired-end reads.

### 4.7 scRNAseq data processing

The gene expression profiles were generated from the raw reads using CeleScope (v1.5.2, Singleron Biotechnologies) with default parameters. Briefly, the barcodes and unique molecular identifiers (UMIs) for each gene-cell combination were extracted and corrected from R1 reads. Adapter sequences and poly A tails were trimmed from R2 reads, which were then aligned against the GRCm38 (mm10) transcriptome using STAR (v2.6.1b). Uniquely mapped reads were assigned to genes using Feature-Counts (v2.0.1). Reads with identical cell barcode, UMI, and gene information were grouped together to create a gene expression matrix for further analysis.

Quality control (QC) and clustering analyses were performed using Scanpy (v1.8.1) under Python 3.7 (40). Cells that expressed less than 200 genes or had fewer than 5 cells expressing a particular gene were excluded in order to filter out low-quality cells based on three metrics: 1) genes expressed in less than 5 cells; 2) cells expressing less than 200 genes; 3) cells with more than 50% mitochondrial gene expression. A total of 72,304 high-quality cells with an average of 1085 genes per cell remained for downstream analyses after filtering steps.

Raw counts data was normalized and transformed into logarithmic scales before selecting the top variable genes by setting flavor = ‘seurat’ among the top-ranked differentially expressed genes. The first twenty principal components identified through Principle Component Analysis (PCA), based on scaled expression profiles, were used for unsupervised clustering utilizing the Louvain algorithm with a resolution parameter set at 1.2. Cell clusters obtained from clustering analysis were visualized using Uniform Manifold Approximation and Projection (UMAP).

### 4.8 Differentially expressed genes analysis

The differentially expressed genes (DEGs) were identified using the Scanpy function “sc.tl.rank_genes_group” based on the Wilcoxon rank sum test with default parameters. Genes with a p-value of ≤0.05 and a log_2_ fold change (log_2_FC) ≥1 or ≤ -1 were considered significantly up- or down-regulated.

### 4.9 Cell type annotation

The major cell types were identified based on the expression of canonical markers obtained from the reference database SynEcoSysTM (Singleron Biotechnology). SynEcoSysTM encompasses a comprehensive collection of canonical cell type markers derived from CellMakerDB, PanglaoDB, and recently published literature for single-cell sequencing data (41). An additional round of clustering was conducted within major cell types, followed by a comparative analysis of their global gene expression patterns with previously identified murine macrophage and CD8^+^ T cell gene signatures from scRNAseq studies (23, 42).

### 4.10 Pathway enrichment analysis

The potential functions of each subcluster were investigated using Gene Ontology (GO) analysis, implemented with the R package “clusterProfiler” (v 3.16.1) (43). The significantly enriched pathways were determined based on a significance level of p<0.05. Bar plots were generated to visualize the selected significant pathways. GO gene sets representing biological processes (BPs) were used as the reference for pathway analysis.

### 4.11 UCell gene set scoring

To elucidate the functional properties of each subcluster of macrophages and neutrophils, gene sets curated from relevant literature were compiled, followed by computation of signature scores for specific gene sets (30, 32, 44–68). The genes corresponding to each score are listed in **Supplementary Table S1**. Gene set scoring was performed using the R package UCell (v 1.1.0) (69). The UCell method is a rank-based scoring approach that is well-suited for analyzing large datasets with multiple samples and batches.

### 4.12 Cell-cell interaction analysis

Cell-cell interactions (CCIs) involving macrophages and CD4^+^ T cells, macrophages and endothelial cells, as well as CD8^+^ T cells and endothelial cells were predicted based on known ligand-receptor pairs using Cellphone DB (v2.1.0) (70). The permutation number for calculating the null distribution of average ligand-receptor pair expression in randomized cell identities was set to 1000. Individual ligand or receptor expression was thresholded using a cutoff based on the average log gene expression distribution across each cell type. Predicted interaction pairs with a p-value < 0.05 and an average log expression > 0.1 were considered statistically significant and visualized using heatmap_plot and dot_plot in CellphoneDB.

### 4.13 RNA velocity

The analysis of RNA velocity was performed using velocyto (v 0.2.3) and scVelo (v0.17.17) in python with default parameters, utilizing BAM files containing macrophages and neutrophils respectively, along with the reference genome GRCm38 (mm10) (71, 72). The resulting data was projected onto the UMAP plot derived from Seurat clustering analysis to ensure visual consistency in visualization.

### 4.14 scATACseq data processing and clustering

Cells with low quality were filtered out based on TSS enrichment < 4 and nFrag < 1,000, while bin regions overlapping with ENCODE Blacklist regions were excluded from downstream analysis. Subsequently, dimensionality reduction was performed using the iterative latent semantic indexing (LSI) approach through the ArchR (v1.0.1.) addIterativeLSI function (73, 74). The ArchR “addClusters()” function was utilized for performing cell clustering. UMAP projection was conducted following the aforementioned procedure.

### 4.15 Integration of scRNA and scATAC data

The integration of scRNAseq data was performed using Seurat’s integration framework to identify corresponding cell pairs between two modalities of data. In this step, the scRNAseq data served as the reference dataset for training the classifier, and each scATAC cell was assigned a cell type based on its similarity to scRNA cells. Specifically, the “FindTransferAnchors” function (reduction = ‘pcaproject’) was employed to identify shared correlation patterns between scATACseq gene activity and scRNAseq gene expression. Subsequently, the “TransferData” function predicted the cell type label for each cell in the scATACseq data. After filtering, a total of 30,139 cells were retained. The filtered scATACseq objects underwent reprocessing with LSI and clustering using SLM algorithm. To assess consistency between predicted cell identities through label transfer and curated annotations based on known marker gene activities, we utilized Jaccard index.

### 4.16 Peaks calling

The peaks were identified by MACS2 based on the aggregated insertion sites from all cells of each cell type. A consensus set of uniform-length non-overlapping peaks was obtained by selecting the peak with the highest score from each set of overlapping peaks. In brief, the significance of peaks was ranked and only the most significant peak was retained for further analysis, while any directly overlapping peak was excluded. This process was repeated iteratively until no more peaks remained.

### 4.17 Motif enrichment analysis

The ArchR “getMarkerFeatures()” function was utilized to obtain differential peaks between two clusters. Subsequently, the “addMotifAnnotations()” function followed by the “peakAnnoEnrichment()” function was employed to calculate transcription factor (TF) motif enrichment in these differential peaks. The JASPAR2020 motif dataset was applied in the “addMotifAnnotations()” function to determine the presence of motifs in the peak set. Finally, the “addDeviationsMatrix()” function was used to compute TF activity enrichment on a per-cell basis across all motif annotations based on chromVAR.

### 4.18 Venn diagram

Differential peaks between Nlrp1b macrophages (Nlrp1b Mac) and other macrophages were filtered based on a log_2_FC threshold of 0.125 and an adjusted P value < 0.05, while overlapping peaks between the two clusters were calculated using Bedtools (v2.31.0) and visualized in a Venn diagram as previously described.

### 4.19 scATAC gene score analysis

The gene scores, which estimate the gene expression and TF motif activity based on scATACseq data, were computed using the “addGeneScoreMatrix()” function with gene score models implemented in ArchR. Subsequently, the gene scores matrix was employed to identify differentially expressed genes, following a similar approach utilized in scRNAseq data analysis.

### 4.20 Statistics

Statistical tests were described in the Methods section. Nonparametric tests in bar graphs were analyzed using a two-sided Student’s t-test with Benjamini-Hochberg correction at P < 0.05. Differential gene expression analysis used an adjusted P < 0.05 cutoff and absolute (average log_2_FC) > 0.25 threshold. Data distribution was assumed to be normal but not formally tested, and no data were excluded from analyses. Blinding was not performed due to cage labeling requirements; data analysis focused on quantitative rather than qualitative measures. For scRNAseq and scATACseq, 19,000-26,000 cells per sample were collected from pools of 4~5 mice as biological replicates.

### 4.21 Study approval

All mouse experiments were conducted in accordance with the guidelines of the Institutional Animal Care and Use Committees of the Third Military Medical University.

## Data Availability

The accession number for the scRNAseq and scATACseq data generated in this study is GSA: CRA019499.
