# Corrigendum: Integrated temporal transcriptional and epigenetic single-cell analysis reveals the intrarenal immune characteristics in an early-stage model of IgA nephropathy during its acute injury

**DOI:** 10.3389/fimmu.2025.1580100

**Published:** 2025-03-07

**Authors:** Chen Xu, Yiwei Zhang, Jian Zhou, Jiangnan Zhang, Hui Dong, Xiangmei Chen, Yi Tian, Yuzhang Wu

**Affiliations:** ^1^ Institute of Immunology, Third Military Medical University (Army Medical University), Chongqing, China; ^2^ The Second Affiliated Hospital, Third Military Medical University (Army Medical University), Chongqing, China; ^3^ Department of Nephrology, Chinese People’s Liberation Army (PLA) General Hospital, Chinese People’s Liberation Army (PLA) Institute of Nephrology, National Key Laboratory of Kidney Diseases, National Clinical Research Center for Kidney Diseases, Beijing, China; ^4^ Chongqing International Institute for Immunology, Chongqing, China

**Keywords:** IgA nephropathy, immune cells, single-cell RNA-seq, single-cell ATAC-seq, macrophages, CD8^+^ T cells

In the published article, there was an error in the **Data availability statement**. The accession number for the scRNAseq and scATACseq data generated in this study was incorrectly displayed as “GSA: CRA024006”. The correct **Data availability statement** appears below.

“The accession number for the scRNAseq and scATACseq data generated in this study is GSA: CRA019499.”

The authors apologize for this error and state that this does not change the scientific conclusions of the article in any way. The original article has been updated.

