# Supplementary material for: Integrated temporal transcriptional and epigenetic single-cell analysis reveals the intrarenal immune characteristics in an early-stage model of IgA nephropathy during its acute injury
Source: Front Immunol. 2024 Oct 18;15:1405748. doi: 10.3389/fimmu.2024.1405748 (PMC11528150; doi:10.3389/fimmu.2024.1405748)
Supplement: Supplementary file 1 [file DataSheet1.pdf]

Supplementary Material

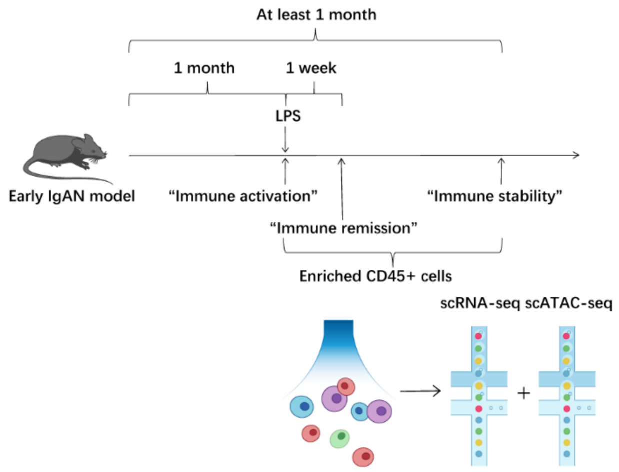

Supplementary Figure 1. Detailed experimental schematic.

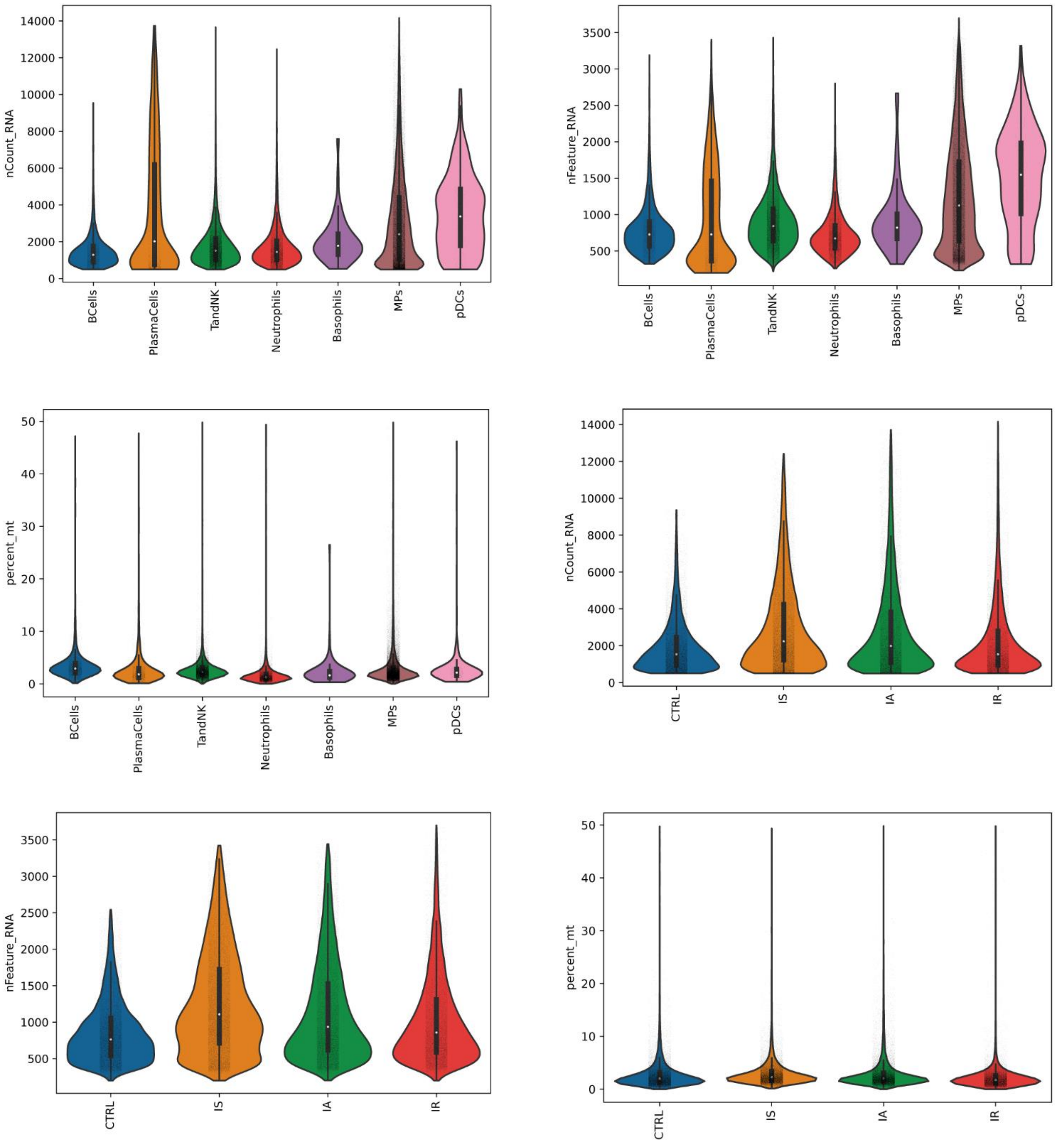

Supplementary Figure 2. Quality control (QC) of scRNAseq data. Number of genes (nCount_RNA), reads (nFeature_RNA) and proportions of mitochondria transcripts (percent_mt) per cell type (upper) and per sample (lower).

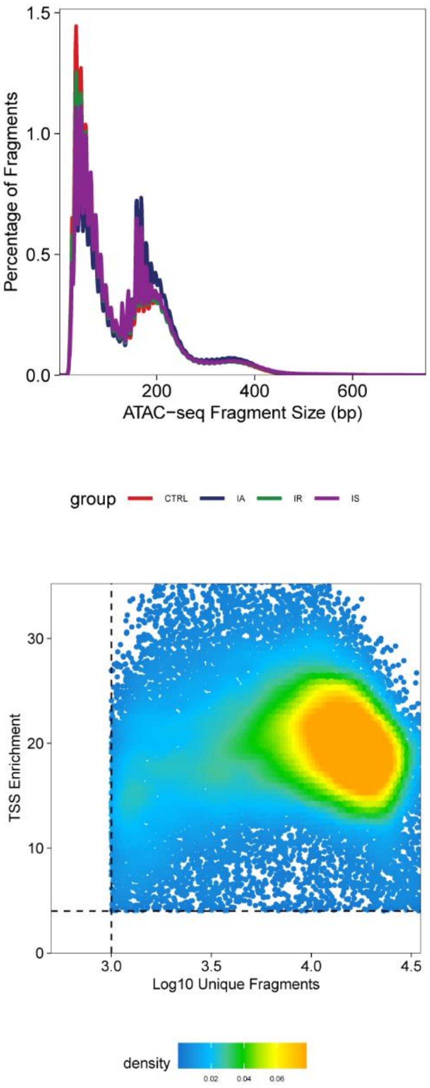

Supplementary Figure 3. QC of scATACseq data. Histogram depicting the normalized read enrichment on the transcription start sites (TSS) of genes from each group. Density plots showing the cells from all groups that passed the TSS enrichment and Log10 unique fragment count threshold.

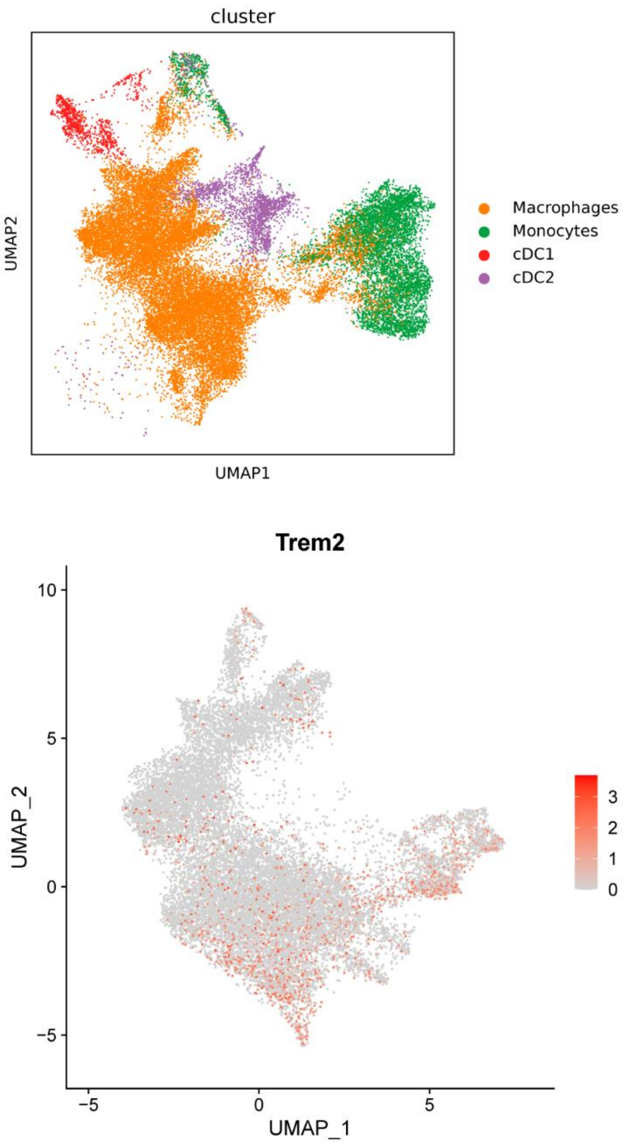

Supplementary Figure 4. UMAP showing the distribution of MPs and Trem2 expression of macrophage. The cells are marked by color code based on the cell type.

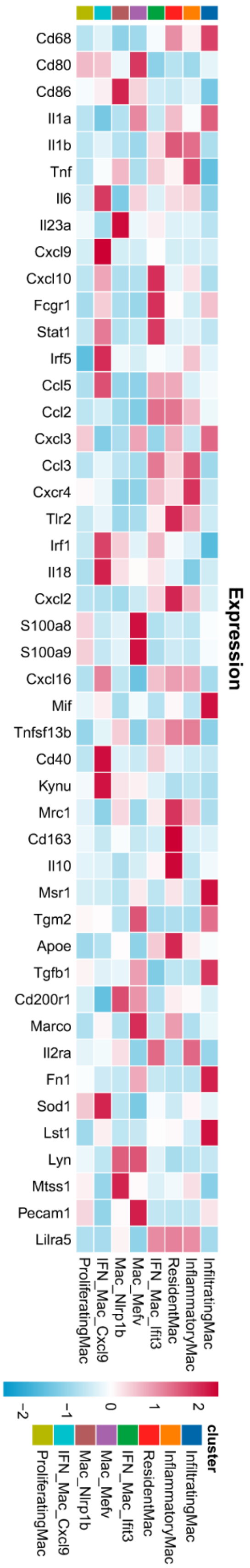

Cd68
Cd80
Cd86
Il1a
Il1b
Tnf
Il6
Il23a
Cxcl9
Cxcl10
Fcgr1
Stat1
Irf5
Ccl5
Ccl2
Cxcl3
Ccl3
Cxcr4
Tlr2
Irf1
Il18
Cxcl2
S100a8
S100a9
Cxcl16
Mif
Tnfsf13b
Cd40
Kynu
Mrc1
Cd163
Il10
Msr1
Tgm2
Apoe
Tgfb1
Cd200r1
Marco
Il2ra
Fn1
Sod1
Lst1
Lyn
Mtss1
Pecam1
Lilra5
ProliferatingMac
IFN_Mac_Cxcl9
Mac_Nlrp1b
Mac_Mefv
IFN_Mac_Ifit3
ResidentMac
InflammatoryMac
InfiltratingMac
Expression
−2
−1
0
1
2
cluster
InfiltratingMac
InflammatoryMac
ResidentMac
IFN_Mac_Ifit3
Mac_Mefv
Mac_Nlrp1b
IFN_Mac_Cxcl9
ProliferatingMac

Supplementary Figure 5. Heatmap of scaled marker gene expression of M1 macrophages (Cd68, Cd80, Cd86, Il1a, Il1b, Tnf, Il6, Il23a, Cxcl9, Cxcl10, Fcgr1, Stat1, Irf5, Ccl5, Ccl2, Cxcl3, Ccl3, Cxcr4, Tlr2, Irf1, Il18, Cxcl2, S100a8, S100a9, Cxcl16, Mif, Tnfsf13b, Cd40, Kynu), M2 macrophages (Mrc1, Cd163, Adora3, Il10, Msr1, Tgm2, Apoe, Tgfb1, Cd200r1, Marco, Il2ra, Fn1), and scaled immunoregulatory genes (Sod1, Lst1, Lyn, Mtss1, Pecam1 and Lilra5) in macrophage subsets.

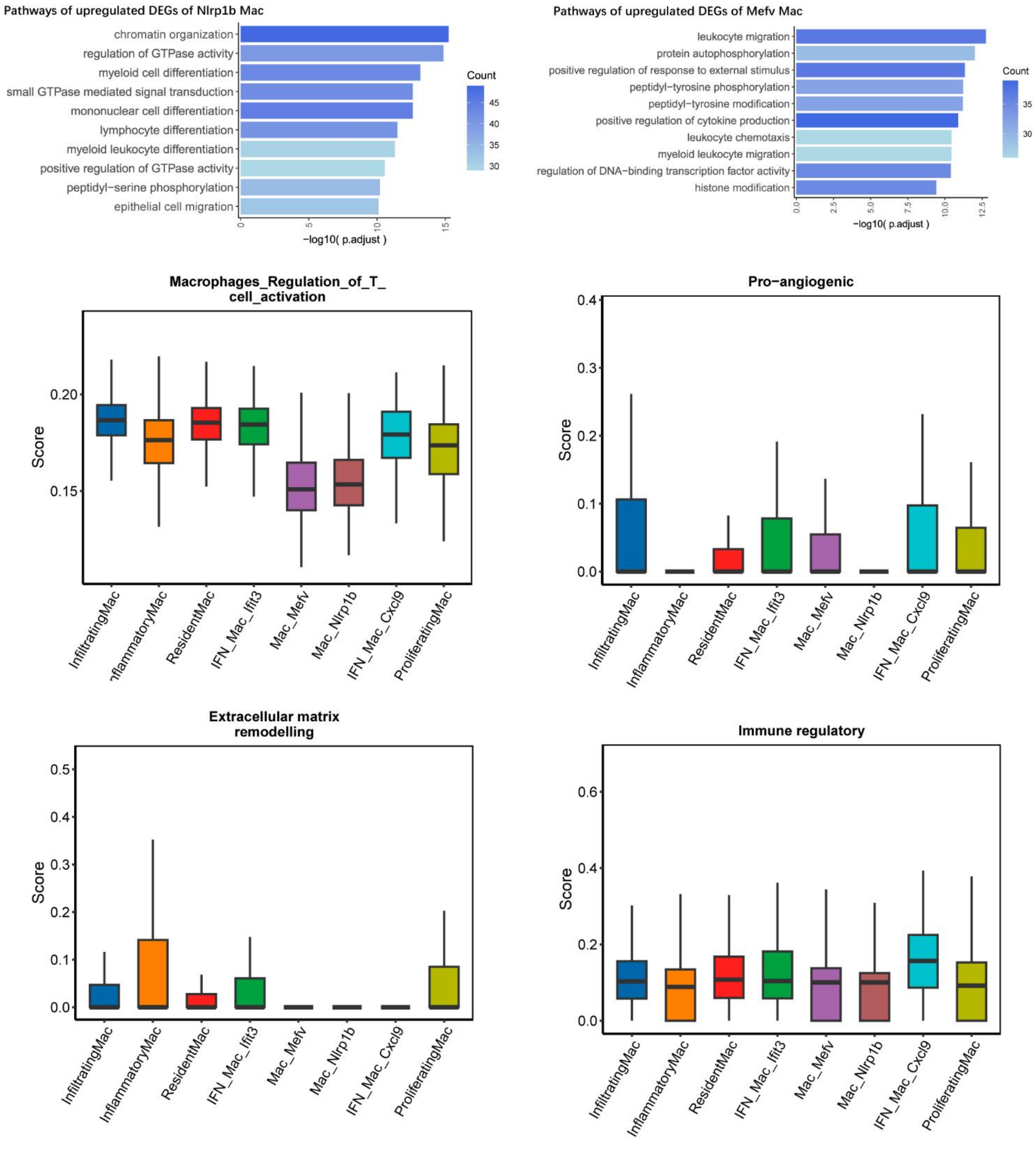

Supplementary Figure 6. Barplots showing enriched pathways of upregulated DEGs of Nlrp1b Mac and Mefv Mac. Boxplot showing the indicated functional scores of macrophage subsets.

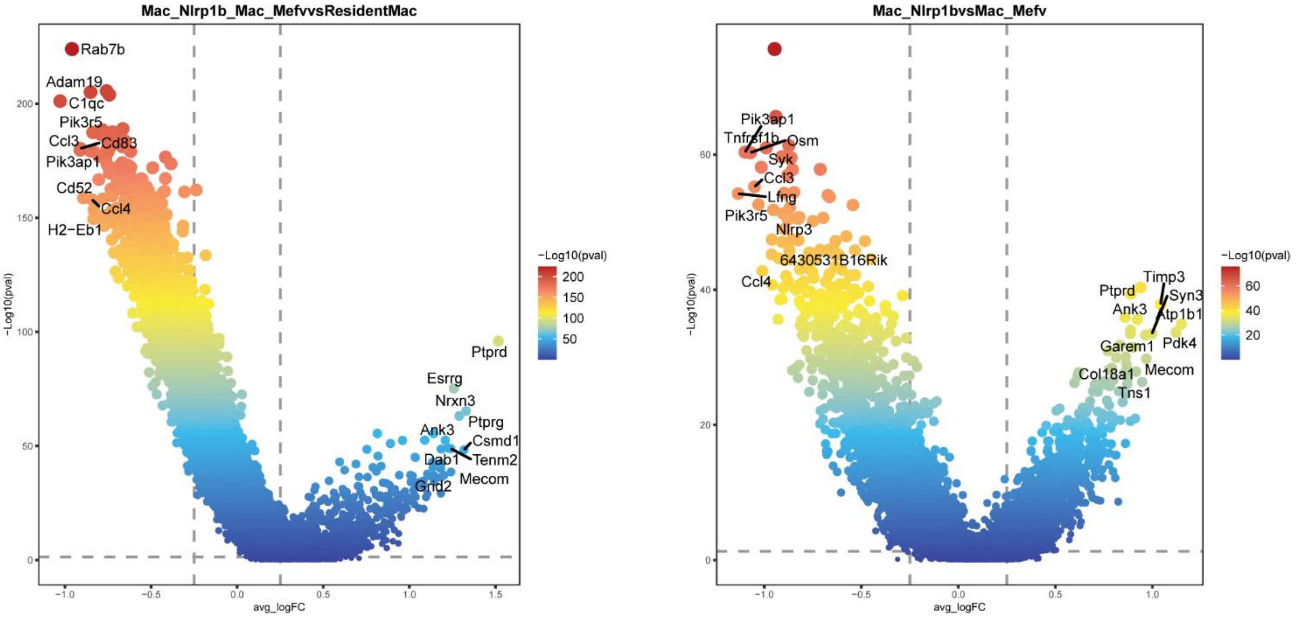

Supplementary Figure 7. Volcano plots showing differential gene activity between indicated macrophage populations.

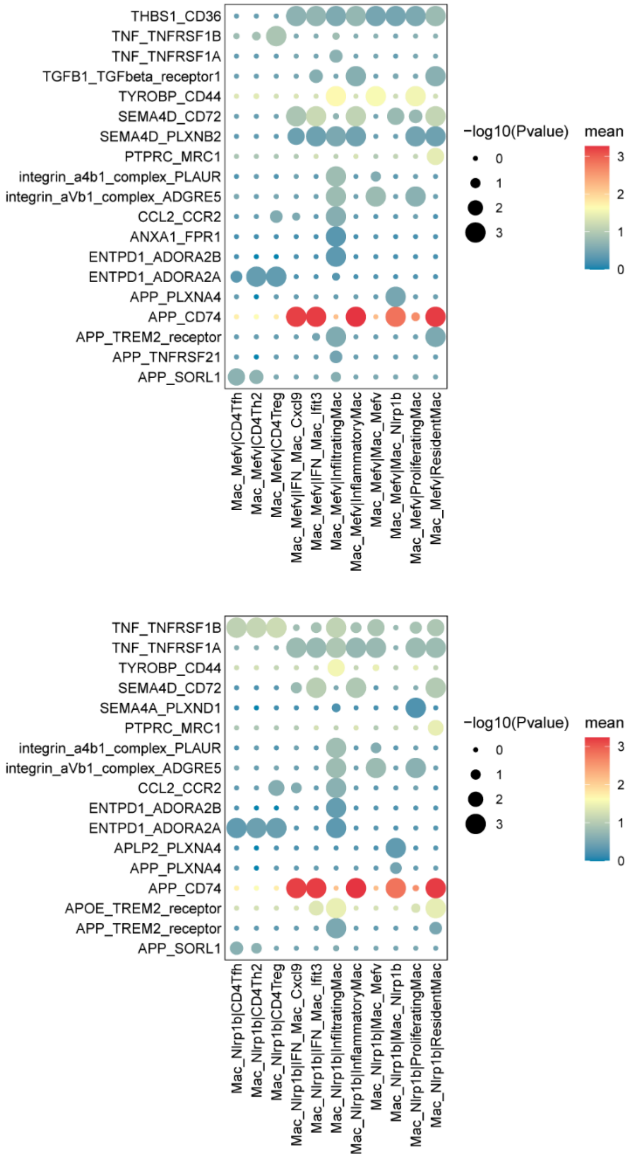

Supplementary Figure 8. Bubble heatmap showing the potential L-R pairs between indicated cells.

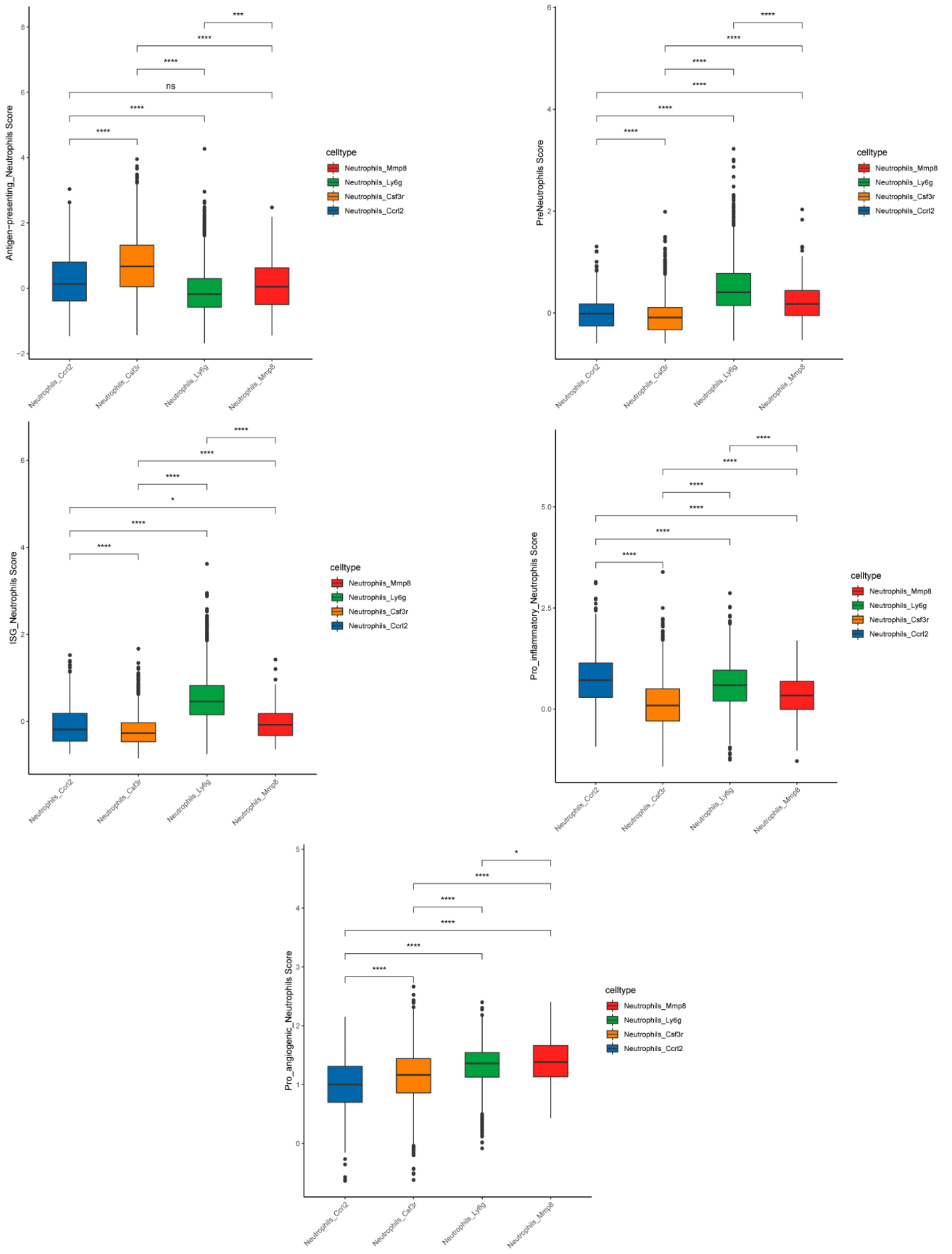

Supplementary Figure 9. Boxplot showing the indicated functional scores of neutrophil subsets.

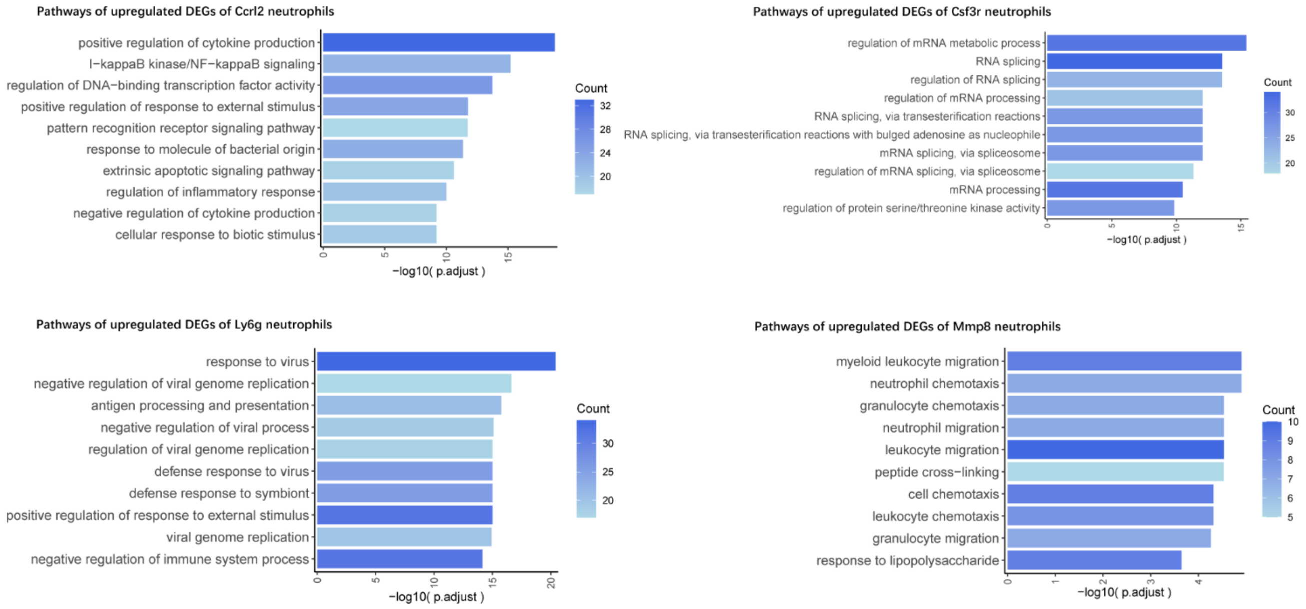

Supplementary Figure 10. Barplots showing enriched pathways of upregulated DEGs of each neutrophil subset.

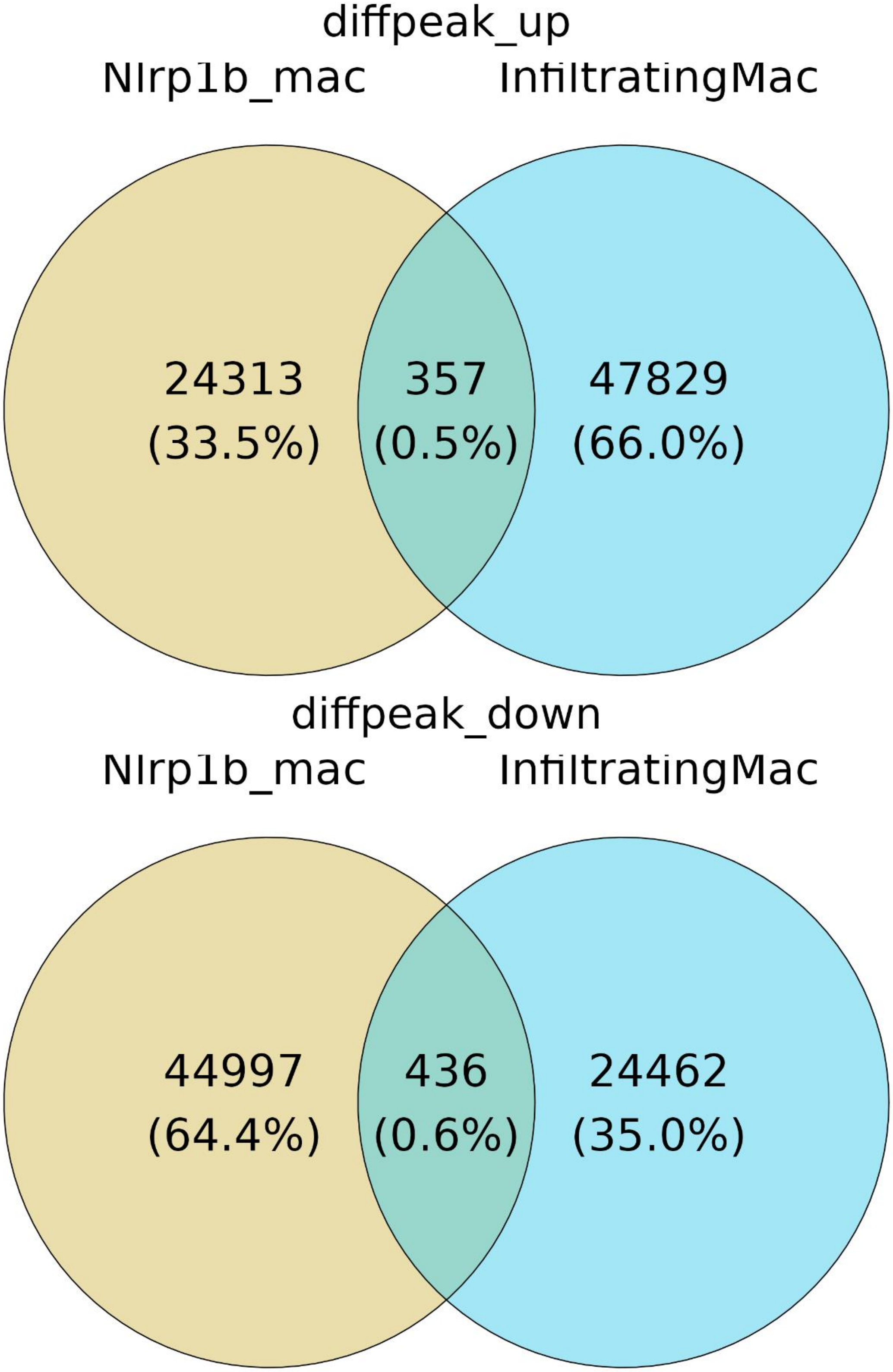

Supplementary Figure 11. Venn diagram of overlapping DACRs in scATAC-seq Nlrp1b Mac and other macrophage clusters.

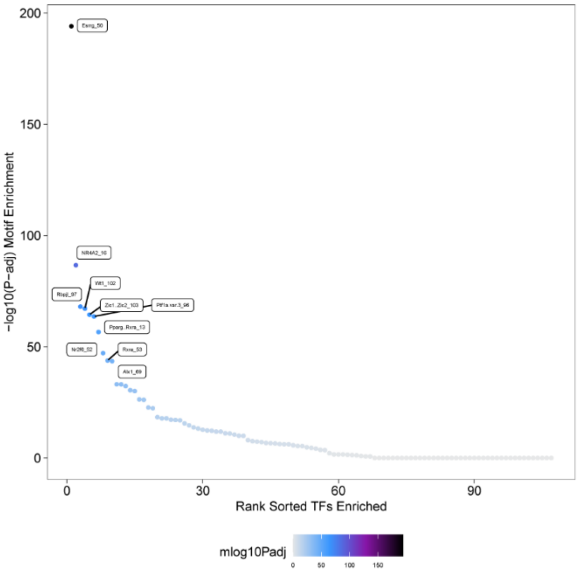

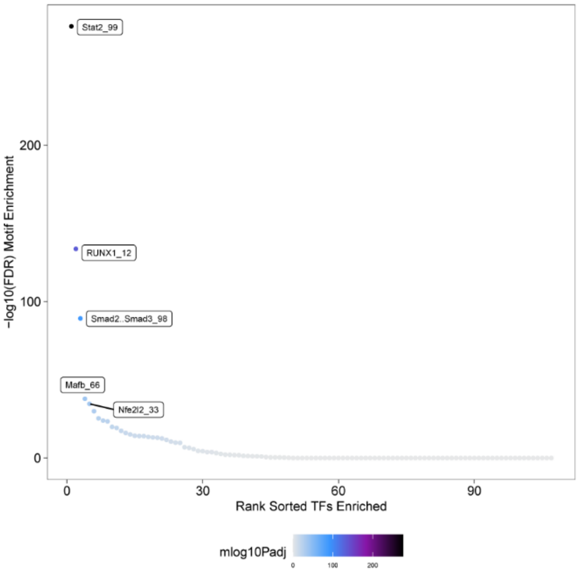

Supplementary Figure 12. Up- (C) and down-regulated (D) TF motif enrichment in DACRs comparing scATAC-seq Nlrp1b Mac and other macrophage clusters.

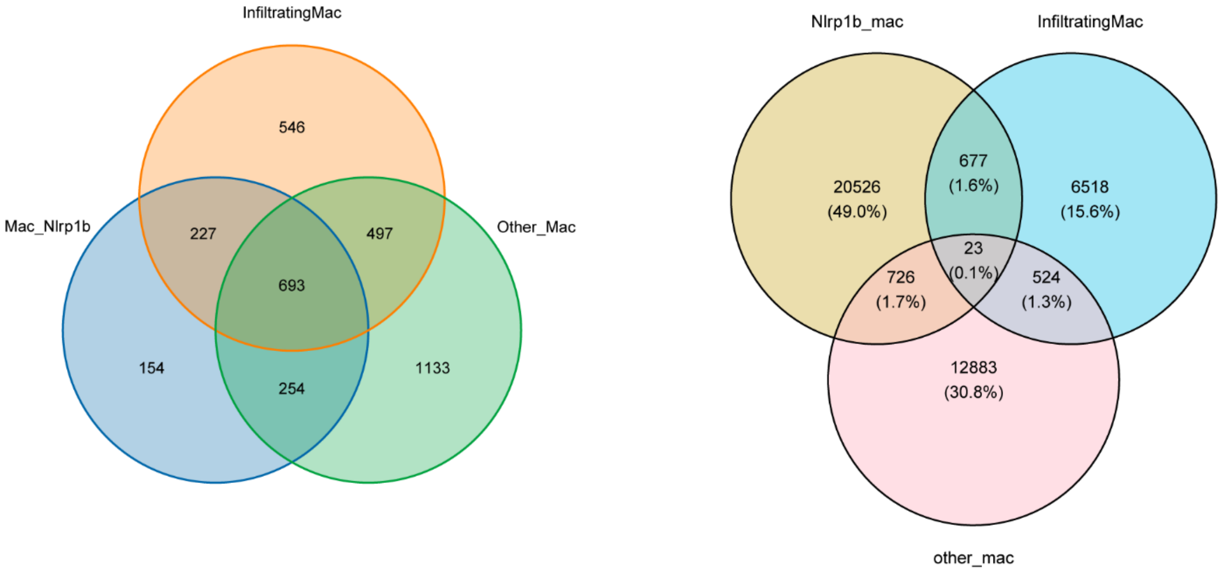

Supplementary Figure 13. Venn diagram of Nlrp1b Mac and other macrophage DEGs, or overlapping DACRs in scATAC-seq Nlrp1b Mac and other macrophage clusters.

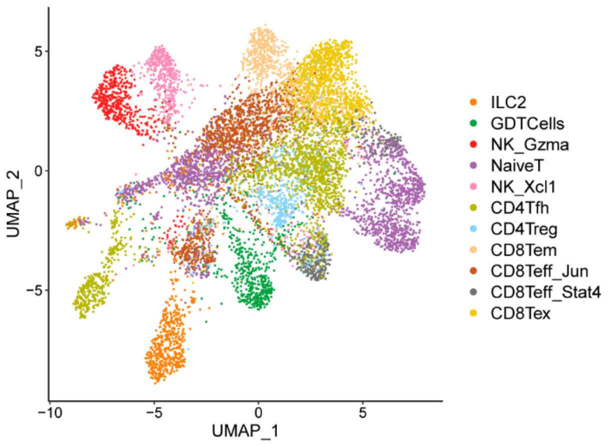

UMAP_1
UMAP_2
ILC2
GDTCells
NK_Gzma
NaiveT
NK_Xcl1
CD4Tfh
CD4Treg
CD8Tem
CD8Teff_Jun
CD8Teff_Stat4
CD8Tex

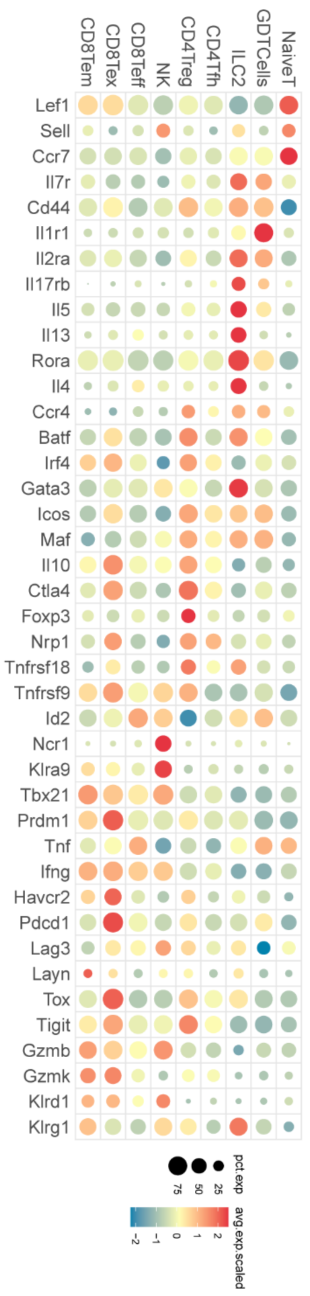

CD8Tem
CD8Tex
CD8Teff
NK
CD4Treg
CD4Tfh
ILC2
GDTCells
NaiveT
Lef1
Sell
Ccr7
Il7r
Cd44
Il1r1
Il2ra
Il17rb
Il5
Il13
Rora
Il4
Ccr4
Batf
Irf4
Gata3
Icos
Maf
Il10
Ctla4
Foxp3
Nrp1
Tnfrsf18
Tnfrsf9
Id2
Ncr1
Klra9
Tbx21
Prdm1
Tnf
Ifng
Havcr2
Pdcd1
Lag3
Layn
Tox
Tigit
Gzmb
Gzmk
Klrd1
Klrg1
pct.exp
avg.exp.scaled

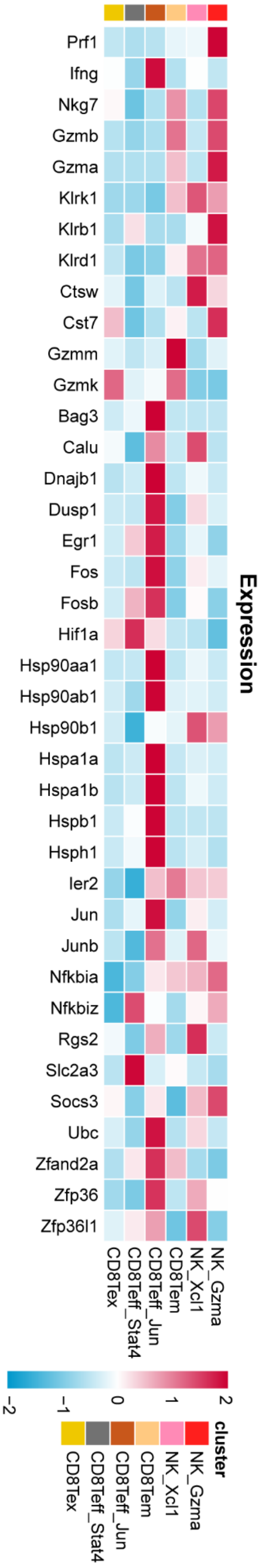

Prf1
Ifng
Nkg7
Gzmb
Gzma
Klrk1
Klrb1
Klrd1
Ctsw
Cst7
Gzmm
Gzmk
Bag3
Calu
Dnajb1
Dusp1
Egr1
Fos
Fosb
Hif1a
Hsp90aa1
Hsp90ab1
Hsp90b1
Hspa1a
Hspa1b
Hspb1
Hsph1
Ier2
Jun
Junb
Nfkbia
Nfkbiz
Rgs2
Slc2a3
Socs3
Ubc
Zfand2a
Zfp36
Zfp36l1
Expression
CD8Tex
CD8Teff_Stat4
CD8Teff_Jun
CD8Tem
NK_Xcl1
NK_Gzma
cluster

Supplementary Figure 14. UMAP of T and NK cells from scATACseq colored by cell type. Dot plot of selected average gene expression values (log scale) and percentage of cells expressing these genes within each cluster. Heatmap of scale cytotoxic genes (Prf1, Ifng, Gnly, Nkg7, Gzmb, Gzma, Gzmh, Klrk1, Klrb1, Klrd1, Ctsw, Cst7, Gzmm, Gzmk) and general stress-associated genes (Bag3, Calu, Dnajb1, Dusp1, Egr1, Fos, Fosb, Hif1a, Hsp90aa1, Hsp90ab1, Hsp90b1, Hspa1a, Hspa1b, Hspa6, Hspb1, Hsph1, Ier2, Jun, Junb, Nfkbia, Nfkbiz, Rgs2, Slc2a3, Socs3, Ubc, Zfand2a, Zfp36, Zfp36l1) per scRNAseq cluster.

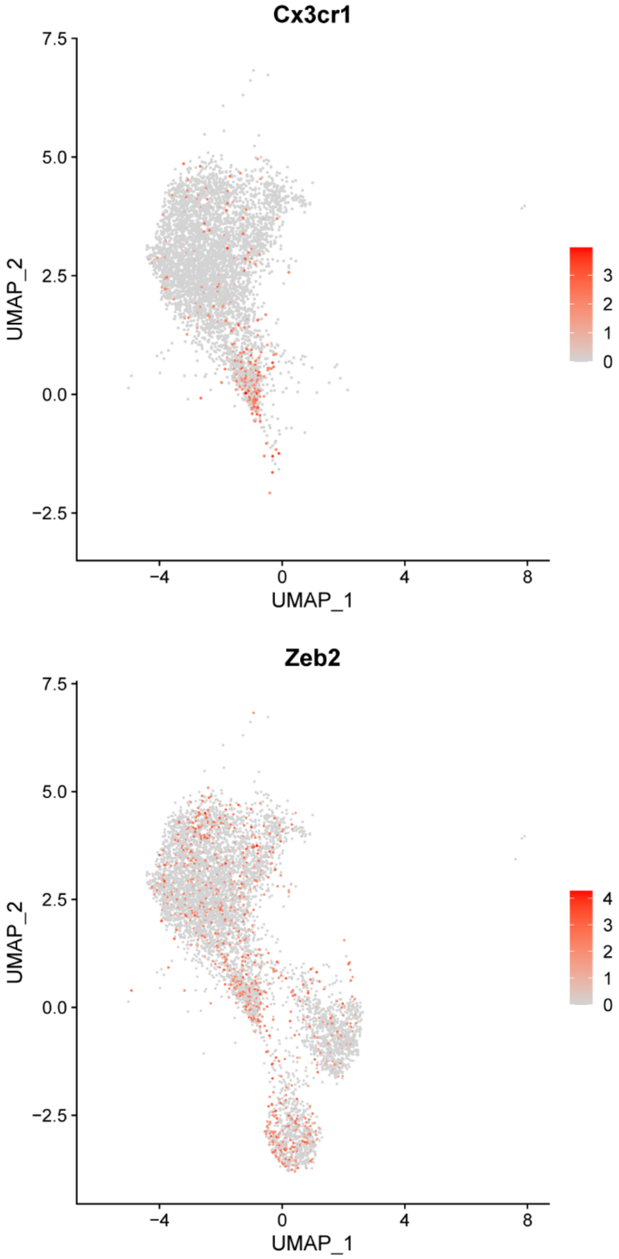

Supplementary Figure 15. UMAP showing the Cx3cr1 expression of scRNAseq CD8+ T-cell subsets and Zeb2 of CD8+ T-cell and NK-cell subsets.
